# A Bioinspired Self‐Healing Conductive Hydrogel Promoting Peripheral Nerve Regeneration

**DOI:** 10.1002/advs.202302519

**Published:** 2023-08-23

**Authors:** Hongyun Xuan, Shuyuan Wu, Yan Jin, Shuo Wei, Feng Xiong, Ye Xue, Biyun Li, Yumin Yang, Huihua Yuan

**Affiliations:** ^1^ School of Life Sciences Nantong University Nantong Jiangsu 226019 P. R. China; ^2^ Key Laboratory of Neuroregeneration of Jiangsu and Ministry of Education Nantong University Nantong Jiangsu 226001 P. R. China; ^3^ Co‐innovation Center of Neuroregeneration Nantong University Nantong Jiangsu 226001 P. R. China; ^4^ NMPA Key Laboratory for Research and Evaluation of Tissue Engineering Technology Products Nantong University Nantong Jiangsu 226001 P. R. China

**Keywords:** cell–matrix interactions, mechanism, peripheral nerve regeneration, self‐healing conductive hydrogel

## Abstract

The development of self‐healing conductive hydrogels is critical in electroactive nerve tissue engineering. Typical conductive materials such as polypyrrole (PPy) are commonly used to fabricate artificial nerve conduits. Moreover, the field of tissue engineering has advanced toward the use of products such as hyaluronic acid (HA) hydrogels. Although HA‐modified PPy films are prepared for various biological applications, the cell–matrix interaction mechanisms remain poorly understood; furthermore, there are no reports on HA‐modified PPy‐injectable self‐healing hydrogels for peripheral nerve repair. Therefore, in this study, a self‐healing electroconductive hydrogel (HASPy) from HA, cystamine (Cys), and pyrrole‐1‐propionic acid (Py‐COOH), with injectability, biodegradability, biocompatibility, and nerve‐regenerative capacity is constructed. The hydrogel directly targets interleukin 17 receptor A (IL‐17RA) and promotes the expression of genes and proteins relevant to Schwann cell myelination mainly by activating the interleukin 17 (IL‐17) signaling pathway. The hydrogel is injected directly into the rat sciatic nerve‐crush injury sites to investigate its capacity for nerve regeneration in vivo and is found to promote functional recovery and remyelination. This study may help in understanding the mechanism of cell–matrix interactions and provide new insights into the potential use of HASPy hydrogel as an advanced scaffold for neural regeneration.

## Graphical abstract

Here, a self‐healing electroconductive hydrogel (HASPy) from HA, cystamine (Cys), and pyrrole‐1‐propionic acid (Py‐COOH), with injectability, biodegradability, biocompatibility, and nerve‐regenerative capacity is constructed. The hydrogel promotes the expression of genes and proteins relevant to Schwann cell (SC) myelination mainly by activating the interleukin 17 (IL‐17) signaling pathway.

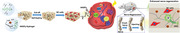

## 1 Introduction

Peripheral nerve injuries (PNI) occur frequently and are a severe clinical condition that can lead to motor or sensory dysfunction, affecting the overall quality of life.^[^ ^1^ ^]^ Nerve regeneration is a challenge owing to limited self‐repair capacity.^[^ ^2^ ^]^ Nerve tissues are sensitive to electrical signals, which can control nerve cell behavior and improve the efficiency of nerve regeneration.^[^ ^3^ ^]^ Therefore, conductive scaffolds can play an essential role in tissue engineering. Typical conductive materials such as polypyrrole (PPy), polyaniline (PANI), polythiophene (PTh), and poly(3,4‐ethylenedioxythiophene) (PEDOT) are commonly used to fabricate artificial nerve conduits.^[^ ^4a,b,c^ ^]^ PANI is inexpensive and easy to obtain and has good electrical conductivity and electromagnetic microwave absorption properties. Compared with PPy, however, PANI is not easy to control in experimental operation and easily causes agglomeration. PTh and its derivatives have excellent environmental stability and good mechanical strength, but the conductivity is weak. The use of PEDOT polymer is limited due to its non‐biodegradability and high stiffness. Therefore, PPy has been extensively studied owing to its high conductivity, environmental stability, and low cytotoxicity. The non‐degradability, brittleness, and poor solubility of PPy^[^ ^5^ ^]^ necessitate the development of modified PPy materials to meet the requirements of nerve regeneration.

Hydrogels are 3D polymer networks that are similar in structure to soft tissue and have drawn extensive attention in the field of tissue engineering.^[^ ^6^ ^]^ Biocompatible conductive PPy hydrogels based on natural extracellular matrix (ECM) components and conductive polymers may function as potential candidates for nerve tissue engineering scaffolds.^[^ ^3^ ^]^ Notably, the lifetime of a conductive PPy hydrogel is shortened by deformation or damage.^[^ ^7^ ^]^ In recent years, self‐healing hydrogels have attracted considerable attention in the field of tissue engineering.^[^ ^8^ ^]^ The network structure and function of a self‐healing hydrogel can self‐repair after damage to improve its lifespan. Hyaluronic acid (HA) is a major ECM component that is widely used in tissue engineering.^[^ ^9^ ^]^ Although HA‐modified PPy films have been prepared for various biological applications,^[^ ^10^ ^]^ the cell–matrix interaction mechanisms are still poorly understood. Moreover, there have been no reports on HA‐modified PPy‐injectable self‐healing hydrogels for peripheral nerve repair.

**Figure 1:**
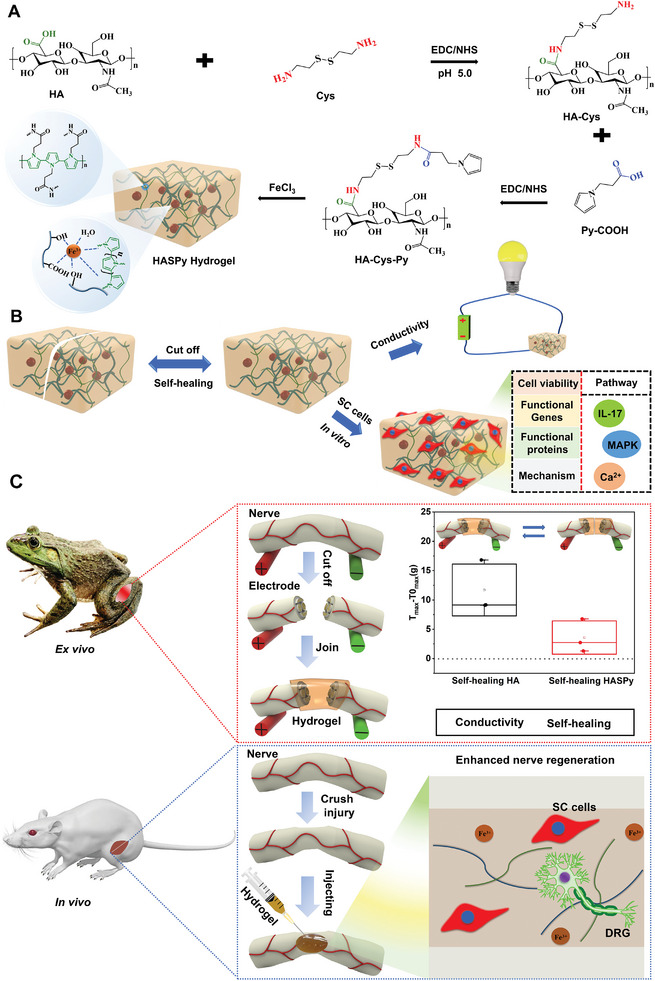
Schematic illustration of HASPy hydrogel construction and application. A) Schematic diagram of the preparation of an injectable self‐healing conductive hydrogel. B) Schematic of the self‐healing and electrical conductivity of the hydrogel that can regulate the expression of Schwann functional genes and proteins. C) Schematic representation of the bullfrog sciatic nerve that can be replaced into hydrogel ex vivo and the rat peripheral nerve that can be regenerated in vivo by injection.

In this study, an injectable self‐healing conductive hydrogel (**Figure** **1**) was prepared using sodium hyaluronate, cysteamine dihydrochloride (Cys), and pyrro‐1‐propionic acid (Py‐COOH). Inspired by the disconnection in the spatial structure of poly‐peptide chains by disulfide bonds in vivo, we introduced Cys disulfide bonds to crosslink HA and Py‐COOH; this endowed the hydrogel with tunable biodegradability under the extracellular matrix microenvironment conditions. Cys was grafted onto HA to form amino‐bond‐functionalized HA (HA‐Cys). Py‐COOH was then grafted onto HA‐Cys by reacting with the amino bond to form Py‐grafted HA (HA‐Cys‐Py). Finally, pyrrole was polymerized by the metal coordination bond of ferric chloride,^[^ ^11^ ^]^ which significantly improved the mechanical properties and conductivity of the HASPy hydrogel. We assessed the physicochemical, mechanical, self‐healing, and electrical conductivity properties of HASPy hydrogel and demonstrated its excellent biological self‐healing and electrical conductivity properties in ex vivo experiments in sciatic nerve–gastrocnemius pairs isolated from bullfrogs. Furthermore, we studied the feasibility of HASPy hydrogel as a nerve substitute in vivo. We also conducted in vitro studies of Schwann cells and in vivo animal experiments to evaluate the effectiveness of the HASPy hydrogel in peripheral nervous system regeneration and the specific underlying mechanism.

## 2 Results

### 2.1 Physicochemical Characterization of HASPy Hydrogel

**Figure 2:**
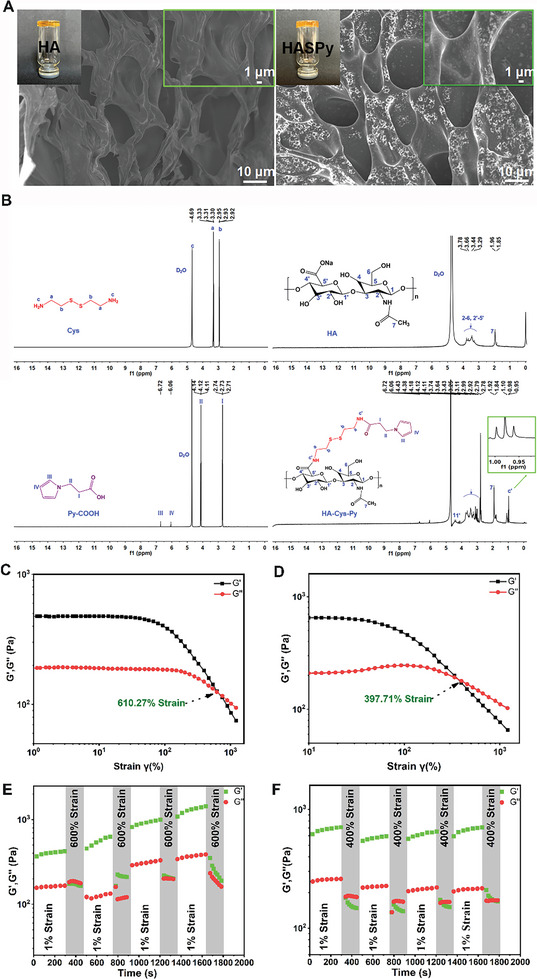
Physicochemical characterization of hydrogels. A) Digital photos of and SEM images of the HA and HASPy hydrogels. B) ^1^HNMR spectra of Cys, HA, Py‐COOH, and HA‐Cys‐Py. C) Strain amplitude sweep analysis of the HA hydrogels, where the crossing points of *G′*, *G″* are their largest strain. D) Strain amplitude sweep analysis of the HASPy hydrogels. E) Thixotropic properties of the self‐healing HA hydrogel at changed strains of 1% and 600%, respectively. F) Thixotropic properties of the self‐healing HASPy hydrogel at changed strains of 1% and 400%, respectively.

**Figure** **2**A shows digital photos of HA and HASPy hydrogels and their microstructures observed using a scanning electron microscope (SEM). The pores of the HA hydrogel are not uniform and do not exhibit their typical homogeneous structure. On the contrary, the porous structure of the HASPy hydrogel is closely proportioned and exhibits a network structure. Furthermore, this hydrogel can be injected through the syringe with a needle (Figure S1, Supporting Information).

The HA‐Cys‐Py was synthesized by stem grafting HA onto small molecules in a two‐step process. The successful preparation of HA‐Cys‐Py was confirmed using ^1^HNMR spectroscopy (Figure 2B). The proton signals of Cys ranged from 3.30 to 3.33 and 2.92 to 2.95 ppm, which were related to methylene at *a* and *b*, respectively, but the proton signal of amino was not visible. HA shows chemical shifts at δ = 3.20–3.69 and 1.85–1.96 ppm, which were responsible for the ring protons (H2–H6, H2′–H5′) and methyl carbon protons (H7), respectively.^[^ ^13^ ^]^ Furthermore, the peaks of the pyrrole ring in Py‐COOH were 6.06 and 6.72 ppm, respectively, and the peaks in δ = 2.71–2.74 and 4.11–4.14 ppm were attributed to methylene protons. After coupling with HA‐Cys, the shift of methylene protons in the pyrrole (peak III and IV, Figure 2B) was slightly reduced. In the HA‐Cys‐Py ^1^HNMR spectrum, the chemical shift between δ = 3.11, 3.25, 2.79–2.99, and 1.84–1.92 ppm was caused by the ectopic protons (H1, H1“), cyclic protons (H2–H6 and H2 “–H5”), and methyl protons (H7) of the HA unit. There were three new chemical shifts in HA‐Cys‐Py between δ = 0.94–1.02 ppm, which were attributed to the proton formation of c” and confirmed the graft of Cys and Py‐COOH to the HA carboxyl group to form amide bonds. In addition, the proton peak of the pyrrole ring appeared in HA‐Cys‐Py, and the signal of the peak was weakened, indicating the successful grafting of Py. Based on the Py‐COOH, Cys, and HA ^1^HNMR spectra (Figure 2B), the grafting of Py‐COOH and Cys could be identified in the ^1^HNMR of the synthesized HA‐Cys‐Py copolymer. This was also confirmed by the appearance of a new absorption peak at ≈1730 cm^−1^ amide (C═O stretching) for HA‐Cys‐Py in the FTIR spectra (Figure S2A, Supporting Information). The absorption peak at ≈1730 cm^−1^ remained and increased in the HASPy hydrogel, but the adsorption peak at ≈1560 cm^−1^ (N─H vibrations) for HA‐Cys‐Py disappeared (Figure S2A, Supporting Information). This phenomenon was due to the formation of ionic covalent coordination crosslinking reactions and hydrogen bonding interactions in the HASPy hydrogel. The polymerization reaction of HA‐Cys‐Py was determined by UV–vis absorption spectroscopy (Figure S2B, Supporting Information). It showed increased absorbance at 460 nm in the HASPy, indicating the polymerization of HA‐Cys‐Py. The content of carboxyl groups in HA‐Cys‐Py was measured by the conductivity titration method (Figure S3, Supporting Information). The grafting ratio of HA‐Cys‐Py was ≈73.82%.

The hydrogel dynamics were measured by assessing the storage modulus (*G′*) and loss modulus (*G″*) of the hydrogel over stain.^[^ ^12^ ^]^ Figure 2C,D shows that *G′* was lower than *G″* at the initial stage, with the curves intersecting at 610.27% and 397.71%, respectively, which represent critical strain values of HA and HASPy hydrogels; this indicates that hydrogels can withstand large elastic deformation. After a high strain, both the HA and HASPy hydrogel networks were destroyed, and the storage modulus *G′* was smaller than the loss modulus *G″*. To further characterize the mechanical properties and self‐healing ability of the hydrogel, the strain of the rheological test was alternated four times between 1% and 600% or 400% of the maximum tolerated strain (Figure 2E,F). The duration of the 1% strain was 290 s, whereas the maximum strain duration was 110 s. When the strain was 1%, *G′* exceeded *G″* and maintained a duration of 290 s, indicating that the structure of the hydrogel was stable at this time. However, when the stress reached 600% or 400%, *G′* was lower than *G″*, indicating that the hydrogel structure was damaged. When the stress was restored to 1%, the HASPy hydrogel recovered (*G′* > *G″*); however, after the maximum stress of the HA hydrogel reached 600%, *G′* was slightly lower than *G″* in the first cycle, while *G′* remained higher than *G″* from second cycle and did not return to the initial state, indicating that the HA hydrogel had a poor self‐healing ability. The transition behavior between 1% and 400% strains of the HASPy hydrogel can still be reproduced after repeated rheological tests. This indicates the remarkable self‐healing ability and mechanical strength of the hydrogel. However, the *G′* and *G″* of the HA hydrogel increased, perhaps owing to increased water loss, thus further toughening the hydrogel.

**Figure 3:**
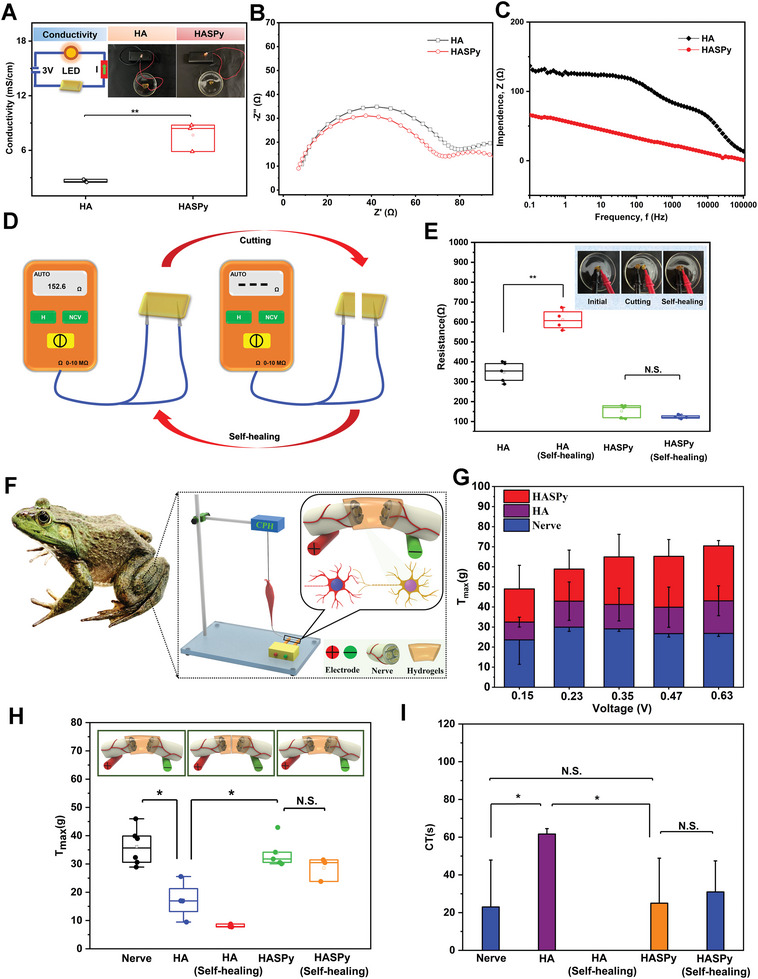
Self‐healing electrical conductivity properties of hydrogels and replacement of bullfrog sciatic nerves by HASPy hydrogel ex vivo. A) The conductivity of HA and HASPy hydrogels (*p < 0.05, **p < 0.01, *n* = 3). B) Nyquist curves. C) Impedance spectra of HA and HASPy hydrogels. D) Schematic diagram of testing hydrogel cutting and self‐healing resistance. E) Resistance comparison of HA and HASPy hydrogels before and after self‐healing (*p < 0.05, **p < 0.01, *n* = 4). F) Schematic diagram of sciatic nerve replaced by HASPy hydrogel was accompanied by gastrocnemius contraction after electrical stimulation. G) The maximal tension T of the sciatic nerve replaced by HA hydrogel and the sciatic nerve replaced by HASPy hydrogel under different voltage stimulation (*p < 0.05, **p < 0.01, *n* = 3). H) Maximum tension of the gastrocnemius muscle after voltage stimulation of the original sciatic nerve, the sciatic nerve replaced by HA, self‐healing HA, HASPy, and self‐healing HASPy (*p < 0.05, **p < 0.01, *n* = 6). I) Nerve conductive time (CT) of the sciatic nerve, the sciatic nerve replaced by HA, self‐healing HA, HASPy, and self‐healing HASPy (*p < 0.05, **p < 0.01, *n* = 3).

The conductivity of the HA and HASPy hydrogels are 2.6 ± 0.17 and 7.7 ± 1.5 mS cm^−1^, respectively (**Figure** **3**A). Importantly, the conductivity of HASPy hydrogels is higher than that of previously reported HA‐based self‐healing conductive hydrogels.^[^ ^14^ ^]^ Figure 3B shows the Nyquist curves of HA and HASPy hydrogels. The diameter of the semi‐circle in the HASPy impedance spectrum is smaller than that of the HA hydrogel, indicating that the impedance of the HASPy hydrogel is smaller. The impedance of the HA and HASPy hydrogels are ≈137 and ≈66 Ω, respectively (Figure 3C). To further investigate the electrical self‐healing of the HA and HASPy hydrogels, they were connected to a resistance meter (Figure 3D). The HASPy hydrogel was observed to self‐heal even when completely disconnected (Figure 3E). This indicates that the HASPy hydrogel has remarkable conductivity and sensitivity, and suggests a positive therapeutic effect on implantation as a replacement for the sciatic nerve.^[^ ^15^ ^]^

The HASPy hydrogel can be stretched to approximately five times its original length, indicating superior flexibility and stretching ability. Moreover, it can still be stretched to ≈4.5 times the original length after repair, showing excellent self‐healing performance. In contrast, HA hydrogels can stretch only to approximately double their original length and have no self‐healing ability, as shown in Figure S4A (Supporting Information). The mechanical properties of the hydrogels were evaluated using a tensile tester. The highest elasticity was achieved for the HASPy hydrogel, and the maximum stress of the fracture was more than double that of the HA hydrogel (Figure S4B, Supporting Information), which was 1.4 times higher than the previously reported HA‐based self‐healing conductive hydrogel.^[^ ^16^ ^]^

The sciatic nerve and gastrocnemius muscle isolated from bullfrogs were selected to verify whether HASPy has good electrical conductivity, which can determine its suitability for implantable nerve replacement. Isolation of the sciatic nerve in bullfrogs to study nerve signaling is based on the principle that an action potential causes the gastrocnemius muscle to contract when the stimulation voltage exceeds the resting potential threshold. The experimental setup is shown in Figure 3F. The positive and negative stimulation electrodes were fixed at the two ends of the sciatic nerve. Stimulated by an additional voltage recorded by a tension sensor, gastrocnemius contraction was clearly observed. In addition, when the normal sciatic nerve was stimulated by gradually increasing the voltage, the degree of gastrocnemius contraction increased correspondingly, and the tension exhibited the same trend, as shown in Figure 3G.

When the intact sciatic nerve was stimulated by voltage, the maximum gastrocnemius tension reached 36.1 ± 6.6 g and disappeared completely after the sciatic nerve was severed (Figure 3H). After connecting the two ends of the severed nerve with HASPy, the normal contraction was re‐established in the gastrocnemius muscle, and no significant difference was observed between the maximum tension of the HASPy‐connected and intact sciatic nerves. Conversely, the HA‐connected sciatic nerve could not reach the maximum tension of the intact nerve. The maximum tension of the HA hydrogel after self‐healing was much smaller than that of a normal nerve, while the maximum tension of the HASPy hydrogel after self‐healing was not significantly different from that of the original state. In addition, we calculated the time required from beginning the voltage application to reaching maximum tension (Figure 3I) and found that the conduction time (CT) measured after HASPy application was not significantly different from that of the intact sciatic nerve, whereas it took longer with HA, suggesting that the conductivity of HASPy is similar to that of the normal nerve. The CT after HASPy self‐repair also showed no significant difference from the original state, indicating good self‐repair recombination of the HASPy hydrogel. Of note, the CT value of the self‐healing HA hydrogel is not shown in Figure 3I because the electrical stimulation does not show a significant tension response, with only the basal value appearing. This proves that HASPy can replace damaged nerves and effectively restore secondary sensory functions, indicating that HASPy had a more optimized self‐repairing performance and that its conductivity could be restored, which is beneficial for nerve repair in vivo. These results indicate the applicability of HASPy as a nerve substitute in vivo.^[^ ^17^ ^]^

**Figure 4:**
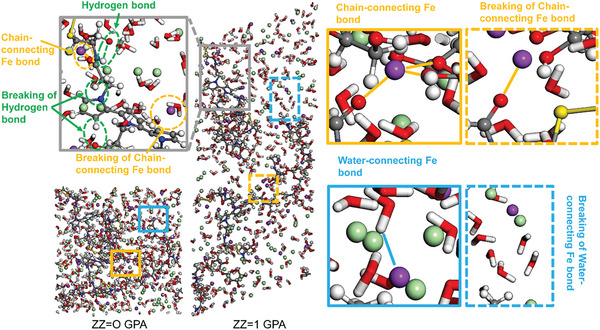
Molecular dynamics simulations of HASPy hydrogels.

Hydrogen bonding and ionic interactions can be demonstrated by molecular dynamics simulations. In Figure S5 (Supporting Information), a few hydrogen bonds were formed between HASPy chains and water molecules. **Figure** **4** illustrates the molecular mechanism of ion improvement in the HASPy‐Water‐Fe^3+^ model, in which Fe^3+^ ions bound the two HASPy chains by the carbonyl oxygens, forming several chain‐connecting Fe bonds. It also shows critical interfacial behavior in which the breaking of chain‐connecting Fe bonds led to higher mechanical properties. This is mainly ascribed to the chain‐connecting Fe bonds confining the disconnection of HASPy chains and facilitating the formation of more hydrogen bonds between HASPy chains. In the process, the breaking of the hydrogen bonds and chain‐connecting Fe bonds lead to noticeably increased energy expenditure. Additionally, the water‐connecting Fe bonds increased the intermolecular forces of regions filled with water molecules. However, the total energy consumption of the fracture of water‐connecting Fe bonds (≈3751.684 kcal mol^−1^) was slightly higher than that of the fracture of hydrogen bonds between water molecules (≈3311.332 kcal mol^−1^). Therefore, the enhancement effect of ions on HASPy chains was obviously greater than that of water molecules.

### 2.2 Degradation and Biocompatibility of HASPy Hydrogel

The in vitro degradation behaviors of the two groups of hydrogels in PBS are shown in Figure S6 (Supporting Information). After soaking in PBS containing dithiothreitol (DTT) for a period, the structure of the hydrogel began to disintegrate, flocs were formed, and the color of the solution became richer. The color gradually turned blue at 4 h, and the solution turned orange again at 36 h. Particularly at 4 h, the structure of the hydrogel became incomplete, indicating that the hydrogel had been degraded. Furthermore, the degradation rate of HASPy in the presence of DTT was faster than that of HA, indicating that DTT influenced the disulfide bond of the HASPy hydrogel. In the pure PBS solution, the degradation rate of the hydrogels in the two groups was not as high as that in the PBS solution with DTT. The structure of the hydrogels began to dissolve after 36 h, and the degradation states of the two groups were similar. As shown in Figure S6 (Supporting Information), compared with the PBS solution, hydrogel degradation in the DTT solution was more thorough and faster when the solution turned green and then yellow. The green color of the solution was attributed to the reduction of ferric chloride to ferrous chloride by DTT,^[^ ^18^ ^]^ whereas the yellow color was attributed to the instability of ferrous chloride, which is oxidized to iron chloride by oxygen in the air. Therefore, the HA hydrogel solution exhibited the same color change. When the hydrogel is in a reducing agent solution, such as DTT, the interior of the cross‐linked disulfide network degrades and collapses, and the structure of the HASPy hydrogel disintegrates from lumps into flocculent structures. The degradation time was controlled by the S─S bond content and DTT concentration.^[^ ^19^ ^]^

To investigate the cytobiocompatibility of the HASPy hydrogel, L929 cells were cocultured with HA hydrogel and HASPy hydrogel for 1 and 3 days. The survival rate of HASPy was 98.9 ± 0.51% at day 1 and 99.43 ± 0.35% at day 3. CCK8 showed that the survival rate of the HASPy group was higher than that of the TCP group. It indicated that HASPy hydrogel had good cytocompatibility (Figure S7A–C, Supporting Information).

To further evaluate the blood compatibility of hydrogels, a hemolysis test was performed. As can be seen from the hemolysis pictures in Figure S7D (Supporting Information), the hemolysis rates of HA and HASPy hydrogels were 1.6 ± 0.09% and 0.5 ± 0.13%, respectively, lower than the international standard (ISO/TR 7405) of 5.0%,^[^ ^20^ ^]^ which verifies the compatibility of the material with red blood cells.

HA and HASPy hydrogels were implanted subcutaneously in rats to evaluate biocompatibility in vivo. First, there was no bleeding or swelling of the skin at the implantation site.^[^ ^21^ ^]^ Hematoxylin and eosin (H&E) staining results at days 7, 14, and 28 are shown in Figure S7E (Supporting Information). As the hydrogel degraded, new tissue was added and the skin structure became increasingly dense. In addition, new blood vessels were observed in the HASPy group, which further ensured the high biocompatibility of the hydrogel. As shown in Figure S7F (Supporting Information), the hydrogel began to degrade on day 14, which was different from the complete hydrogel on day 7. Only black matter remained, indicating that the hydrogel had degraded to a certain extent. Tumor necrosis factor‐α (TNF‐α) was used as a macrophage marker to evaluate the inflammatory response of the hydrogels. No significant difference was observed in fluorescence intensity between the HA and HASPy hydrogels (Figure S7G,H, Supporting Information). Moreover, the fluorescence intensity of the hydrogels decreased significantly on day 14, which may have been due to the degradation of the hydrogels, leading to a decreased inflammatory response. This is the basis for candidate neural replacement materials.

### 2.3 Effects of HASPy on Schwann Cell Growth and Expression of Functional Genes and Proteins

**Figure 5:**
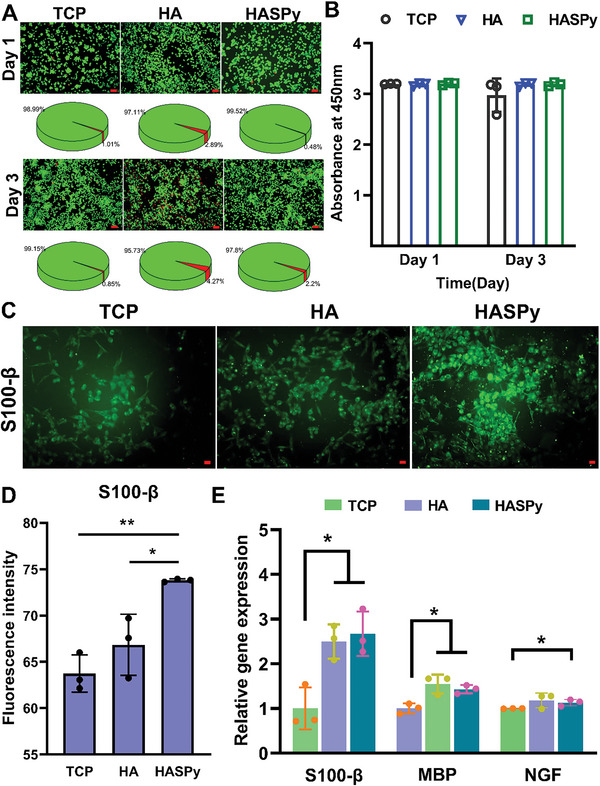
Expression and functional verification of related genes and proteins in Schwann cells on the hydrogel. A) Live/Dead staining and survival rate of Schwann cells cultured on TCP (tissue culture plate), HA, and HASPy hydrogel. Scale bar: 100 µm. B) Cell Counting Kit‐8 (CCK‐8) assay (*p < 0.05, **p < 0.01, *n* = 3). C) Immunofluorescence images of Schwann cells after 3 days of growth in TCP, HA, and HASPy groups. Scale bar: 50 µm. D) Fluorescence intensity of *S100‐β* (*p < 0.05, **p < 0.01, *n* = 3). E) Gene expression of *S100‐β*, *MBP*, *NGF* by qRT‐PCR test (*p < 0.05, **p < 0.01, *n* = 3).

To further evaluate the effects of the HASPy hydrogel on Schwann cells, the most important host cell in the peripheral nerve, HASPy was co‐cultured with Schwann cells for 3 days. As shown in **Figure** **5**A,B, for Schwann cells, no significant difference was observed in cell viability among the HASPy, HA, and Tissue Culture Plate (TCP) groups as well, indicating excellent compatibility. This is because HA, a natural polysaccharide, is an important structure throughout the body and has excellent biocompatibility.^[^ ^22^ ^]^ These results show that the HASPy hydrogel has good cytocompatibility, rendering it a potential material for nerve repair.

To analyze the expression levels of Schwann cell biomarkers glial fibrin *S100‐β*, we proceeded with immunofluorescence staining. Figure 5C shows the staining of Schwann cells cultured on TCP, HA, and HASPy for 3 days. Figure 5D shows the fluorescence intensity of Schwann cells in the TCP, HA, and HASPy groups. The results showed that the expression of *S100‐β* in the HASPy group was significantly higher than that in the TCP and HA groups, indicating that the HASPy hydrogel promoted the expression of Schwann cell‐related functional proteins.

To investigate whether genes related to nerve regeneration were regulated in the hydrogel group, three genes related to myelination, cytoskeleton development, proliferation, and Schwann cells, including myelin basic protein (*MBP*), nerve growth factor (*NGF*), and Schwann cell marker (*S100‐β*), were selected for further RT‐qPCR analysis (Figure 5E). Our results showed that the expression of *MBP* and other genes in Schwann cells on the hydrogels was significantly higher than that in the TCP group. The expression level of *MBP* was higher in the HA and HASPy groups because *MBP* is an important marker of myelination in the early stage, indicating that the hydrogels helped Schwann cells form myelin around axons, further proving that the HA and HASPy hydrogels may be beneficial to myelin formation during nerve regeneration. In this study, compared with the TCP group, the HA and HASPy groups showed relatively high *NGF* release, indicating lower early apoptosis and higher expression levels of *S100‐β*, and thus good physiological function of Schwann cells. Although *S100‐β* expression in the HASPy group was slightly higher than that of the HA group, the difference was not significant.

**Figure 6:**
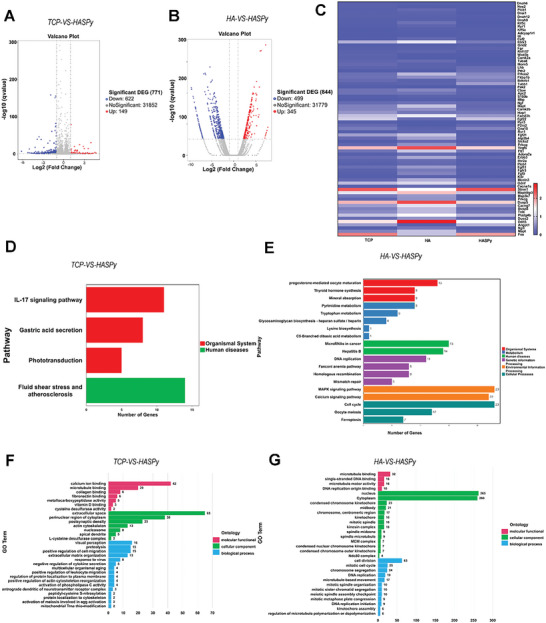
Transcriptomic sequence. A) Volcano map of TCP‐VS‐HASPy. B) Volcano map of HA‐VS‐HASPy. C) Heatmap of differentially expressed genes in TCP, HA, and HASPy groups. D) KEGG pathway enrichment of TCP‐VS‐HASPy. E) KEGG pathway enrichment of HA‐VS‐HASPy. F) GO terms of TCP‐VS‐HASPy. G) GO terms of HA‐VS‐HASPy.

Transcriptomic sequencing generated a volcanic map showing the volcano plot generated 622 down‐regulated and 149 up‐regulated differentially expressed genes in the HASPy group compared to the TCP group (**Figure** **6**A). Compared with those in the HA group, 499 down‐regulated and 345 up‐regulated differentially expressed genes were identified in the HASPy group (Figure 6B). A total of 73 upregulated and downregulated differentially expressed genes were identified. Using the Fragments Per Kilobase Million (FPKM) values of differentially expressed genes under different experimental conditions as expression levels, hierarchical clustering analysis was performed to create a heat map (Figure 6C); 22 genes related to the calcium signaling pathway, 15 genes related to the Mitogen‐Activated Protein Kinase (MAPK) signaling pathway, 5 genes related to the IL‐17 signaling pathway, and 31 genes related to neurogenesis were screened out.

The Kyoto Encyclopedia of Genes and Genomes (KEGG) diagram showed that four pathways were enriched in the HASPy‐VS‐TCP group, and the expression level of the IL‐17 signaling pathway in Schwann cells on HASPy conductive hydrogel was significantly higher than that in other signaling pathways (Figure 6D). KEGG analysis showed that the HA‐VS‐HASPy groups were mainly enriched in the calcium signaling pathway and MAPK signaling pathway (Figure 6E). Gene ontology (GO) analysis showed that enriched GO biological process terms of the TCP‐VS‐HASPy group were related to the generation and regulation of postsynaptic density, actin cytoskeleton, response to virus, and anterograde dendritic neurotransmitter receptor complex (Figure 6F). The GO map of HA‐VS‐HASPy showed enrichment related to cell division, mitotic cell cycle, chromosome segregation, and DNA replication (Figure 6G).

**Figure 7:**
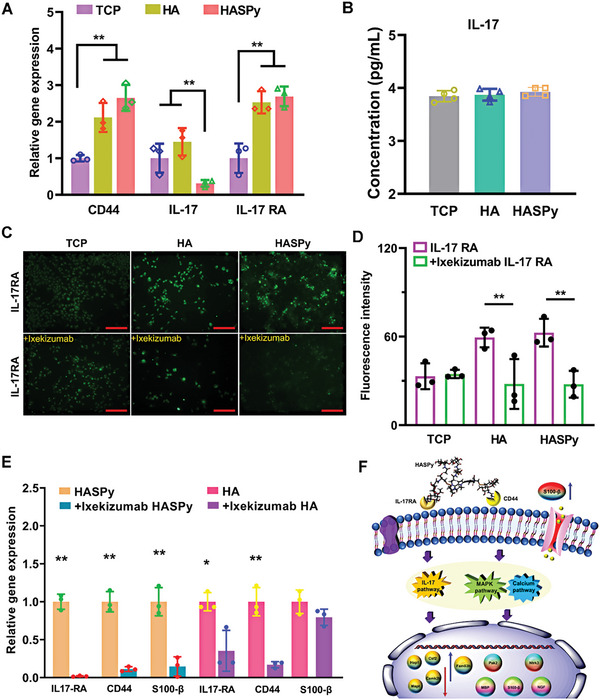
Verification of interaction mechanism between HASPy and Schwann cells. A) Gene expression of *CD44*, *IL‐17* and *IL‐17RA* in TCP, HA and HASPy (*p < 0.05, **p < 0.01, *n* = 3). B) The cytokine secretion of IL‐17 in the supernatant of Schwann cells was detected by ELISA (*p < 0.05, **p < 0.01, *n* = 3). C) Immunofluorescence of Schwann cells in TCP, HA, and HASPy groups before and after inhibitor (up: IL‐17RA; down: +Ixekizumab IL‐17RA). Scale bar: 250 µm. D) Fluorescence intensity of IL‐17RA (*p < 0.05, **p < 0.01, *n* = 3). E) Gene expression of *IL‐17RA*, *CD44*, and *S100‐β* in Schwann cells cultured on HA and HASPy hydrogel with Ixekizumab (*p < 0.05, **p < 0.01, *n* = 3). F) Mechanism summary of HASPy on Schwann cells' behavior.

To explore the triggering mechanism of the HASPy hydrogel on inflammation, we selected CD44 as the research object. HA can interact with the receptor CD44, which promotes inflammation. CD44 was selected for qPCR analysis. As shown in **Figure** **7**A, compared to the TCP and HA groups, the HASPy group showed the highest expression of CD44, indicating that HASPy significantly increased the level of CD44 genes in Schwann cells. This result verified our hypothesis that HASPy is likely to induce an inflammatory response in the presence of HA through CD44. Many cytokines have been found to mediate IL‐17 cytokine responses, most of which use a common receptor subunit, IL‐17RA.^[^ ^23^ ^]^ Gene expression studies have shown that the expression of a large number of genes associated with innate immune cell recruitment to infection or tissue injury sites is promoted by IL‐17RA signal transduction.^[^ ^24^ ^]^ In view of this, the growth of Schwann cells on HASPy hydrogel increased the expression of the IL‐17 pathway; therefore, we selected IL‐17RA to verify the high expression of the IL‐17 pathway. To further explore the activation of the IL‐17 pathway, we assessed the expression levels of IL‐17 and IL‐17RA and found that IL‐17 levels in the HASPy group were significantly lower than those in the TCP and HA groups, indicating that IL‐17 expression level was low. In addition, the IL‐17RA expression level in the HASPy group was higher than that in the TCP and HA groups (Figure 7A). To explore the specific mechanism by which HASPy hydrogel activates the IL‐17 pathway in Schwann cells, we assessed the level of IL‐17 in the supernatant of Schwann cells by ELISA, and the results showed no significant differences among the three groups (Figure 7B). This indicates that HASPy hydrogel promotes the expression of the IL‐17 pathway by binding to IL‐17RA. The expression of IL‐17RA was detected by immunostaining. The results in Figure 7C show that the fluorescence intensity of IL‐17RA in the hydrogel co‐cultured Schwann cells was significantly higher than that in the TCP group. Quantitative results of fluorescence intensity also showed that IL‐17RA was most highly expressed in the HASPy group, indicating that the IL‐17 pathway was highly expressed in the HASPy group (Figure 7D). Further, ixekizumab, the inhibitor of IL‐17RA, was added to verify the expression of IL‐17RA. The immunofluorescence staining diagram of IL‐17RA showed that the number and intensity of fluorescent cells were significantly reduced in the presence of ixekizumab (Figure 7C,D). This indicated that the expression of IL‐17RA was inhibited. Furthermore, the gene expression level of Schwann cells was tested after the addition of the inhibitor. The results showed that after ixekizumab was added, the IL‐17RA level in the HA and HASPy group was significantly lower than that in the TCP group and that the expression level of CD44 was also inhibited, suggesting that IL‐17RA may affect the expression of CD44. The expression levels of Schwann cell function‐related genes, such as *S100‐β*, were also inhibited, as shown in Figure 7E. Related possible mechanism diagrams have also been summarized in Figure 7F.

### 2.4 Molecular Dynamic Simulations

**Figure 8:**
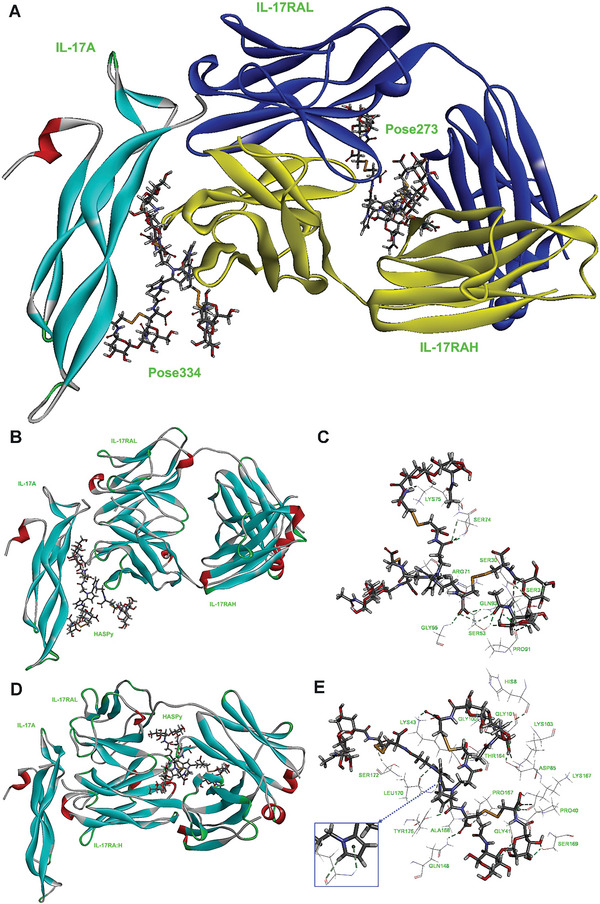
Molecular Dynamic (MD) simulations HASPy hydrogel binding to IL‐17 or IL‐17 receptor. A) Two conformations of pose 334 and pose 273 were chosen according to the score result. Pose 334 and pose 273 were represented by sticks. IL‐17A protein was represented by a solid ribbon. IL‐17RAL protein was represented by a blue solid ribbon. IL‐17RAH protein was represented by a yellow solid ribbon. B) The IL‐17(A, RAH)/HASPy conformation in the last 50 ps of the production simulation. HASPy was represented by a stick. IL‐17A, IL‐17RAL, and IL‐17RAH proteins were represented by solid ribbons. C) The hydrogen bonds between HASPy and residues in IL‐17A compound protein. HASPy and residues were represented by sticks. The hydrogen bonds were shown as green dotted lines. D) The IL‐17RA (H,L)/HASPy conformation in the last 50 ps of the production simulation. HASPy was represented by a stick. IL‐17A, IL‐17RAL, and IL‐17RAH proteins were represented by solid ribbons. E) The hydrogen bonds between HASPy and residues in IL‐17A compound protein. HASPy and residues were represented by sticks. The hydrogen bonds were shown as green dotted lines.

According to the result of the ZDOCK score, pose 334 and pose 273 were the optimal configurations coming from 100 top poses in the largest cluster with scores of 13.68 and 12.66, respectively (**Figure** **8**A). As shown in Figure 8A, pose sites could be located between IL‐17A and IL‐17RAL, H, or between IL‐17RAL and IL‐17RAH. The results indicated that the HASPy micromolecule interacted with both IL‐17A and IL‐17RA and directly interacted with IL‐17RA (L, H). To further investigate the mechanism of interaction, molecular dynamic (MD) simulations were performed for each docked complex to monitor the stability of all backbone atoms of IL‐17A and HASPy micromolecules (Pose 334, Pose 273). After the steepest descent and conjugate gradient minimization, the complexes (pose 334, pose 273) were heated, equilibrated, and finally, production was executed in an explicit solvent environment at a temperature of 310 K and pressure of 1 bar. The production of the system began after equilibration, and heating remained in equilibrium (Figure S8B,C,F,G, Supporting Information) and during the last 50 ps (Figure S8D,H, Supporting Information). In vivo 334, 273 showed that only the structure of the IL‐17A/HASPy compound changed in a physiological saline environment. This result indicates that the 3D model was stable and could be used in further studies. The interaction energy was employed to calculate the energy (interaction, electrostatic interaction, and van der Waals interaction energies) of the 50 conformations from the production of the equalized system (Figures S8E,I, Supporting Information). The conformation with the highest interaction energy was selected for further analyses. Figure 8B displays the optimal conformation of the HASPy and IL‐17A compound protein, where HASPy mainly interacted with IL‐17A and IL‐17RAH. HASPy mainly interacts with IL‐17RA (H, L), as shown in Figure 8D. Both conformations showed strong interactions with HASPy and IL‐17A.

To confirm the most likely conformation, the monitor H Bonds, we calculated the interaction energy (binding energy, hydrophobic residue interactions). Owing to the hydrogen bond and hydrophobic interactions, the two conformations exhibited remarkable total interaction energy, which was calculated to be −201.93 and −245.69 kcal mol^−1^, respectively. As shown in Figure 8C and Table S1 (Supporting Information), nine residues interacting with HASPy formed 21 strong and 1 weak HBonds. The weak HBond originated from H_B1_ in SER74 (IL‐17RAH) and O_35_ in HASPy, the distance and angle DHY of which were 3.0 Å and 90.6°, respectively, while 17 residues interacting with HASPy produced 21 strong and 2 weak HBonds (Figure 8E; Table S5, Supporting Information). Two weak HBonds were produced between H_N_ in GLY41, H_A_ in PRO40 (IL‐17RAL), and O_145_ in HASPy. The distance between the HBonds in H_N_, H_A_, and O_145_ was 2.8 Å, and their angles were 96.4° and 99.7°, respectively. Both weak HBonds were stronger than H_B1_ in SER74 (IL‐17RAH) and O_35_ in HASPy cells. Moreover, N‐H_N_ in LEU170 (IL‐17RAH) and the five‐membered ring in polypyrrole (HASPy) exist as single π‐type hydrogen bonds because the lone pairs on atom N in polypyrrole produce electron conjugate effects (Figure 8E; Table S5, Supporting Information). The single π‐type hydrogen bond causes the hydrogen bond N‐H_N_…HASPy to bend, with a deviation angle of 36.8°. Therefore, the conformation of HASPy interacting with IL‐17RA (H, L) (IL‐17RA (H, L)/HASPy) is more stable than that of HASPy interacting with IL‐17A and IL‐17RAH (IL‐17(A, RAH)/HASPy).

The total interaction energy of IL‐17RA (H, L)/HASPy was higher than that of IL‐17(A, RAH)/HASPy (−245.69 vs −201.93), which could be due to increased hydrophobicity, and their hydrophobic interaction energy was −13.36 and −22.58 kcal mol^−1^, respectively. Residues are considered pivotal in the complex if they bound the ligand with an interaction energy of less than −1 kcal mol^−1^.^[^ ^25^ ^]^ The interaction energies of HASPy and residues at the active site of IL‐17A were calculated and are listed in Tables S3 and S6 (Supporting Information), respectively. Six key residues were VAL90, ILE92, LEU116, VAL119, VAL124, and H_ILE51 in the IL‐17(A, RAH)/HASPy complex and their interaction energy levels were −2.19, −2.75, −5.91, −1.05, −1.18, and −1.28 kcal mol^−1^, respectively. In particular, the LEU116 residue (−5.91 kcal mol^−1^) played an important role in the hydrophobic interaction. In the IL‐17RA (H, L)/HASPy complex, four key residues were H_LEU108, H_ALA168, H_VAL169, and H_LEU170 whose interaction energy were −1.46, −9.15, −2.98, and −7.27 kcal mol^−1^, respectively. The H_ALA168 and H_LEU170 residues contributed significantly more to the hydrophobic interaction than the LEU116 residue. The results showed that the hydrophobic interaction energy of IL‐17RA (H, L)/HASPy was higher than that of IL‐17(A, RAH)/HASPy (−22.58 vs −13.36). This is also consistent with the experimental results, which imply that hydrophobic residues around the binding site of HASPy are beneficial for wrapping HASPy in the active pocket of the IL‐17RA protein. Therefore, the IL‐17RA (H, L)/HASPy conformation was more stable than the IL‐17(A, RAH)/HASPy conformation, which is most likely to exist and is consistent with the experimental results.

### 2.5 Effects of HASPy Hydrogel on Nerve Health and Function

**Figure 9:**
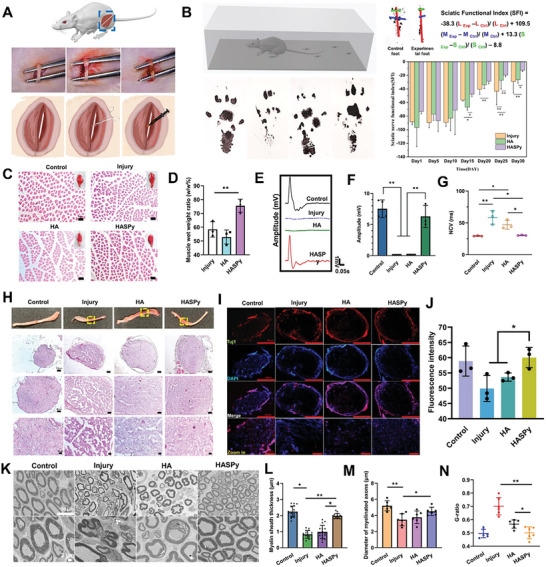
Sciatic nerve repair with hydrogels for 30 days. A) Surgical image of sciatic nerve implanted with HASPy hydrogel in SD rats. Left: Sciatic nerve. Middle: The sciatic nerve is crushed. Right: Injection of HASPy hydrogel. B) Apparatus for rat footprint acquisition. Images of footprints on day 30. The formula of the sciatic nerve functional index. The sciatic nerve functional index of rats from day 1 to day 30 (*p < 0.05, **p < 0.01, *n* = 3). C) H&E staining of gastrocnemius fibers and Scale bar: 100 µm. D) The wet weight ratio of the injured side to the normal side (*p < 0.05, **p < 0.01, *n* = 3). E) Comparison of electrophysiological recordings of complex muscle action potentials (CMAPs). F) Onset‐to‐peak amplitude (*p < 0.05, **p < 0.01, *n* = 3). G) nerve conduction velocities (NCVs) for healthy groups, injured groups and implanted with different hydrogels (*p < 0.05, **p < 0.01, *n* = 3). H) Images of nerves on day 30. H&E staining image of nerve transects. I) Fluorescence images of control, injury, HA, and HASPy group. J) Fluorescence intensity of Tuj1 (*p < 0.05, **p < 0.01, *n* = 3). K) TEM images of cross‐section in four groups. Scale bar: 10 µm (up) and 2 µm (down). L) Myelin sheath thickness (*p < 0.05, **p < 0.01, *n* = 16). M) Diameter of myelinated axons (*p < 0.05, **p < 0.01, *n* = 6), and N) *G*‐ratio (*p < 0.05, **p < 0.01, *n* = 6).

The HASPy hydrogel was implanted into rats for 30 days to determine its effects on nerve health and function, with the HA hydrogel and injury groups as the reference. **Figure** **9**A shows the appearance of the nerve after being crushed, showing a translucent color. After the hydrogel was injected, the color of the squeezed area deepened, indicating successful implantation of the hydrogel. The formula for calculating the sciatic functional index (SFI) is shown in Figure 9B. We set up a narrow passage covered with white paper. The hind paws of the rats were smeared with black ink, and their black footprints on the passage were collected to observe the extent of toe extension. As shown in Figure 9B, the degree of spread of the rat footprints of the HASPy hydrogel group was like that of normal footprints. All rats were in a relatively extended state, indicating that the neural sensory function of the rats was restored. We observed the nerve 30 days after the injury to examine recovery. At 15, 20, 25, and 30 days after injury, rats treated with the HASPy hydrogel had significantly higher SFI scores than rats treated with the neuro‐injected HA hydrogel and injury. The control foot and experimental foot correspond to the left and right feet of the rats, respectively, and the calculation results of the SFI can be obtained using the formula. The SFI was used to test the effect of the hydrogel on functional recovery after a crush injury. SFI is a reliable and widely used method for quantifying animal recovery after sciatic nerve injury by comparing the footprint of the injured side with that of the uninjured control side.^[^ ^26^ ^]^ Sciatic nerve injury causes loss of nerve and muscle function in the foot and reduction in toe extension and middle toe spread, resulting in a longer footprint. As the muscles and nerves recover, the toes become increasingly extended, leading to the formation of a normal footprint. The footprint showed a sequential increase in the degree of extension of the surgical claw, indicating that the degree of neurological function recovery increased over time.^[^ ^2^ ^]^ According to the SFI results in Figure 9B, the HASPy group had a faster recovery rate from day 10 and scored higher than the other two groups. Overall, the injury and HA groups regained 70.9 ± 3.8% and 73.6 ± 6.2% of their original function, respectively, and the HASPy‐treated rats regained ≈87.5 ± 1.8% of their original function within 30 days, suggesting that the HASPy group was closest to normal function. This suggests that the HASPy hydrogel can promote sciatic nerve regeneration most rapidly after crushing. Neurological dysfunction after peripheral nerve injury can lead to atrophy and dysfunction of the innervated gastrocnemius muscle. When the injured nerve function is restored, the gastrocnemius muscle, as the target organ, is innervated again, and muscle atrophy is reduced accordingly. Therefore, morphological recovery of the target muscle can be used as a reliable parameter to evaluate nerve regeneration. Here, the wet‐to‐weight ratio (injured side gastrocnemius/normal side gastrocnemius) and muscle fiber size were measured to validate the therapeutic effect. Thirty days after surgery, H&E staining of the gastrocnemius muscle showed atrophy in all groups. As the gastrocnemius muscle is innervated by the sciatic nerve and its structure and function can reflect the recovery of the nerve, the wet‐to‐weight ratio of the gastrocnemius muscle was examined, and muscle fibers were stained with H&E (Figure 9C).^[^ ^13^ ^]^ The normal gastrocnemius muscle (left) of each group was larger than the gastrocnemius muscle on the operation side (right), indicating significant atrophy of the right gastrocnemius muscle. The muscle morphology of the experimental side in the HASPy group was closest to that of the control side, and the degree of atrophy was relatively low. To quantitatively evaluate muscle atrophy, the muscle weight ratio was calculated from the muscle weight ratio of the injured and control sides (Figure 9D). The gastrocnemius muscle weight ratio was 58.42 ± 5.56% in the injury group, 52.87 ± 5.10% in the HA group, and 75.49 ± 4.77% in the HASPy group. The recovery rate of the gastrocnemius muscle in the HASPy group was significantly higher than that in the injury and HA groups.^[^ ^2^ ^]^

During the experiment, all the rats survived normally without postoperative complications. The gastrocnemius muscle, the terminal target organ innervated by nerves, can directly reflect the recovery of nerve function.^[^ ^27^ ^]^ Thirty days after transplantation, compound muscle action potentials (CMAP) of injured and healthy limbs were collected with electromyograms (EMG) for electrophysiological analysis, which was used to measure the contractile force of the tibialis anterior muscle.^[^ ^13^ ^]^ The amplitude and latency of CMAP were analyzed simultaneously to verify nerve innervation of the gastrocnemius muscle. The larger the signal amplitude, the more optimized the electrical conductivity of the nerve, the more axons with conduction function, the shorter the incubation period, the lower the nerve conduction velocity, and the higher the degree of nerve remyelination.

Figure 9E shows that different CMAP signals were detected in the different experimental groups. The peak amplitudes of CMAP in the control, injury, HA, and HASPy groups were 7.53 ± 1.407, 0.097 ± 0.006, 0.13 ± 0.018, and 6.297 ± 1.774 mV, respectively (Figure 9F). The HASPy and control groups had similar peak shapes, and the control group had the highest average CMAP peak amplitude; the HASPy group was the closest to the control without significant difference, and the injury and HA groups were significantly different from both the control and HASPy groups, indicating that the HASPy group could repair the damaged nerve the fastest and transduce the bioelectrical signals.

In addition, there was no significant difference in nerve conduction velocity (NCV) between the HASPy (30.333 ± 0.577 ms) and control (30.333 ± 0.577 ms) groups, as shown in Figure 9G. The NCV of the injury group (58.333 ± 10.599 ms) and HA group (47 ± 7 ms) was significantly lower than that of the other groups. The average peak amplitude of the CMAP and nerve conduction velocity (NCV) of the HASPy and control groups were not significantly different. The quantitative results showed that HASPy had a higher amplitude and shorter latency than the two other groups, suggesting that HASPy hydrogel can accelerate the recovery of sciatic nerve electrophysiological function.

To evaluate the effects of the HASPy hydrogel on nerve regeneration, we analyzed H&E staining and immunofluorescence images (Figure 9H–J) of the transverse and longitudinal sections of the nerve segment obtained 30 days after implantation. As shown in Figure 9H, the nerves of the three groups in the operation group, injury, HA, and HASPy, all showed tissue hyperplasia and some redness and swelling. Histological sections and immunostaining showed that the cross sections of the nerves in the control group were narrower than those in the other three groups. The regenerated nerve bundles in the HASPy group were dense and orderly, with a thicker epineurium (EP) than those in the injury and HA groups (Figure 9H; Figure S9, Supporting Information).^[^ ^2^, ^28^ ^]^ H&E images also revealed that nerves in the HASPy group could see blood vessels, but not in the injury group, and less so in the HA group. The HASPy group showed significantly promoted nerve regeneration.

The slow growth of axons is one of the biggest challenges in peripheral nerve regeneration; therefore, the speed of axon regeneration has become a key concern in the field of nerve injury. In nerve cells, β‐III‐tubulin protein is mainly expressed in neurons and may be involved in neurogenesis.^[^ ^29^ ^]^ To verify the expression of β‐III‐tubulin in each group, we performed cross‐cutting immunofluorescence staining of the nerve samples. As shown in Figure 9I, the nerve structure of the HASPy group was dense and the myelin sheath structure was intact, whereas the nerve structure of the injury group was sparse and the nerve outer membrane was loose. The HASPy group was denser and exhibited stronger fluorescence than the HA group. For this reason, quantitative statistics of fluorescence intensity were carried out, as shown in Figure 9K; the β‐III‐tubulin expression of the HASPy group showed the highest fluorescence intensity. This indicates that the HASPy group was more significantly involved in nerve regeneration.

*S100‐β* is a glial cell marker and is commonly used to evaluate glial expression.^[^ ^30^ ^]^ Longitudinal sections of the nerve samples were used to stain *S100‐β*. Immunostaining plots showed that *S100‐β*‐positive nerve fibers in the HASPy group had the highest density, largest area, and most compact arrangement compared to the injury and HA groups, and were like those in the control group, as shown in Figure S10 (Supporting Information). The immunofluorescence intensity in the HASPy group was nearly 1/5 higher than that in the injury group. There were significant differences between the two groups. The fluorescence intensity of the HASPy group was higher than that of the HA group. The injury group showed significantly less intensity of positive nerve fibers owing to the relatively low number of glial cells (e.g., Schwann cells) and incomplete recovery of the injured nerve.

Sciatic nerve recovery was assessed by immunohistochemical staining and transmission electron microscopy (TEM) 30 days postoperatively. TEM images of the transverse section (half section) of the middle of the regenerated nerve segment are shown in Figure 9K. The degree of myelination and axon maturation of nerve fibers was further demonstrated using TEM images of the regenerated nerves. The number of axons, the diameter of myelinated nerve fibers, the thickness of myelin sheaths, and the g‐ratio (defined using the inner and outer diameters of the myelin sheaths) were quantified.^[^ ^31^ ^]^ The regenerated nerve fibers in the control and HASPy groups presented good myelination and a dense myelin lamina in Figure 9L. The average diameter of myelinated nerve fibers in the HASPy group was larger than that in the injury and HA groups. However, it did not reach the level of the normal group; there was no significant difference between the diameters of the HASPy and control group fibers, indicating that axons tended to mature (Figure 9M). The average g‐ratio is an important indicator of axonal myelination,^[^ ^32^ ^]^ and Figure 9N shows that the G ratio in the control and HASPy groups were similar; both were larger than those in the injury and HA groups, indicating a thicker myelin sheath, while the g‐ratio in the injury and HA groups was smaller, indicating a lower degree of myelination in the axons. There were significant differences in myelin thickness and g‐ratio among the HASPy, injury, and HA groups, but no differences were observed between the HASPy and control groups. However, HASPy had more myelinated axons than the control group, perhaps due to the larger myelin sheaths in the control group. These results suggest that the HASPy group presented a more positive effect on myelin regeneration in regenerated nerves than the HA group did, and showed a better recovery than that of the injury group.

## 3 Discussion

Peripheral nerve regeneration injury causes profound inconvenience to patients; hence, a solution is urgently required.^[^ ^2^ ^]^ Hydrogels can provide a suitable microenvironment for nerve regeneration.^[^ ^33^ ^]^ In recent years, self‐healing materials have prolonged the service life of materials, reduced replacement costs, and improved system safety.^[^ ^34^ ^]^ Furthermore, conductive materials can promote the growth of neurites and axons.^[^ ^35^ ^]^ An injectable, degradable, self‐healing conductive hydrogel was successfully constructed in this study. HA is derived from the ECM; being a biomaterial, it is biodegradable, biocompatible, and absorbable.^[^ ^9^ ^]^ PPy has become an ideal choice for developing conductive and nerve repair materials because of its convenient synthesis, suitable mechanical properties, and excellent electrical conductivity. However, the poor degradation of PPy limits its development and application.^[^ ^36^ ^]^ Therefore, we chose Py‐COOH to graft onto HA, which successfully solved the problem of its difficult degradation. We selected a Cys‐containing disulfide bond as an intermediate to connect HA to Py‐COOH, and the response of Cys can be obtained by disulfide bonds in vivo.^[^ ^37^ ^]^ Disulfide bonds can initiate chain exchange reactions under exposure to heat, UV light, and redox conditions without any catalysts or initiators.^[^ ^38^ ^]^ The formation of chain‐connecting Fe bonds, hydrogen bonds, and disulfide bonds provide the hydrogel self‐healing properties. Therefore, the hydrogel prepared in this study is not only degradable but also self‐repairing and conductive; this has not been explored in previous nerve regeneration studies. We also systematically evaluated the effects of the biomimetic scaffold on peripheral nerve regeneration using physicochemical analysis, in vitro cell culture, and molecular biology evaluation. The HASPy hydrogel exhibited good conductivity and biocompatibility and could promote the expression of functional proteins in Schwann cells. Further exploration of the application of HASPy hydrogel in sciatic nerve repair in rats showed that HASPy hydrogel was beneficial for nerve regeneration in rats, which is consistent with the Schwann cell study.

In addition, we explored the mechanism of action of the HASPy hydrogel in nerve regeneration via transcriptome sequencing. Transcriptomic sequencing results showed that the HASPy hydrogel activated the IL‐17 signaling pathway in Schwann cells. IL‐17R is widely expressed and can directly act on Schwann cells. We verified the expression of IL‐17RA through qPCR results (Figure 7A), which revealed the activation of the IL‐17 signaling pathway. Previous studies have confirmed that IL‐17RA expression levels can be significantly decreased after IL‐17 overexpression stimulation.^[^ ^39^ ^]^ Our qPCR results showed that the IL‐17 level was significantly downregulated, and the ELISA assay revealed that it was slightly decreased, although the expression level of IL‐17RA was increased. This indicates that the IL‐17 signaling pathway was indeed activated by IL‐17RA and did not increase the expression of IL‐17. The molecular dynamics simulation also demonstrated the possible binding mode of the HASPy hydrogel to IL‐17 or IL‐17RA. The simulation showed that the IL‐17RA (H, L)/HASPy conformation was more stable than that of IL‐17(A, RAH)/HASPy, and more likely to exist, which is consistent with our genetic and protein detection results (Figure 8).

The base material of the HASPy hydrogel is HA; therefore, we focused on analyzing its effect on Schwann cells. It is well known that HA and HA fragments are commonly internalized by cells via CD44.^[^ ^40^ ^]^ CD44 can interact with cell surface‐related growth factors, enzymes, and cytokines to stimulate signal transduction.^[^ ^40^, ^41^ ^]^ Our qPCR results (Figure 7A) showed significantly higher expression of CD44 in the HASPy group. CD44 has been confirmed to activate the key MAPK signaling pathway.^[^ ^40^ ^]^ Transcriptomic sequencing results showed the activation of both the MAPK and calcium signaling pathways. The MAPK signaling pathway plays a role in many biological responses; it is thought to regulate cell survival, proliferation, and differentiation during nerve regeneration,^[^ ^42^ ^]^ and activation of the MAPK pathway (e.g., PAK2) may upregulate the expression of *S100‐β* and other proteins, which is beneficial for myelin regeneration and nerve repair. As MBP is an important marker of early myelination,^[^ ^43^ ^]^ HASPy may be beneficial for myelination during nerve regeneration. NTRK3 is involved in the calcium signaling pathway, and the activation of this pathway is also beneficial for axon extension and guidance to promote nerve regeneration.^[^ ^43^ ^]^ We determined that the enrichment of calcium signaling in the HASPy hydrogel group may be due to the presence of PPy that enhances its electrical conductivity, and thus elicits a calcium signaling response.^[^ ^44^ ^]^

To further explore the possible interaction between CD44 and IL‐17RA, we inhibited the expression of IL‐17RA and found that not only was the expression level of CD44 decreased, but also that a series of Schwann cell‐related genes were downregulated. This may suggest that IL‐17RA acts on CD44, thereby regulating a series of signaling pathways and physiological and biochemical reactions in Schwann cells.

Through transcriptome sequencing, qPCR, and immunostaining, the possible mechanism by which the effluent gel affects the behavior and physiological function of Schwann cells is summarized in Figure 7F. Schwann cells first encountered the hydrogel through different receptors on the cell membrane. Hydrogels may activate receptors on the cell membrane, leading to the activation of the correspondent pathway that upregulates or downregulates the relevant genes or proteins. As shown in Figure 7F, the *Fam83h* gene associated with the IL‐17 signaling pathway was upregulated, and the significantly downregulated genes, such as *Hap1*, *Camk2b, Csf2*, and *Mapt*, were associated with neurodegenerative pathways.

Mechanistic analysis showed that Schwann cells can be activated by HASPy via different signaling pathways, causing changes in genes and proteins related to nerve repair. These results indicate that on the whole, activation of the IL‐17 signaling pathway in Schwann cells is related to the interaction between HASPy and IL‐17RA.

In conclusion, the greatest advantage of the hydrogel scaffold developed here is that it simulates the in vivo environment and integrates injectable capacity, self‐healing, degradation, conductivity, and good mechanical properties, as well as biocompatibility, allowing for the evaluation of hydrogels at the molecular, cellular, and animal levels, respectively. Furthermore, the molecular mechanism of the hydrogel synergistic effect on Schwann cells was addressed, and the interaction between HASPy and Schwann cells was simulated using molecular dynamics. These findings are significant to the study of the synergistic effect of degradable self‐repairing conductive hydrogels on nerve regeneration. Schwann cell behavior regulation should also be clarified further through gene sequencing, inhibition, and molecular biology technology, although their gene and protein expression were clarified in our preliminary assessment. Moreover, the corresponding knockout experiments have not been verified. Meanwhile, the internal mechanism of Schwann cell pathway activation has only been partially discussed, and further research is required. In addition, to better evaluate the effect of the prepared hydrogel on nerve regeneration, the molecular mechanism of our in vivo experiment must be verified. Finally, although the HASPy hydrogel facilitated peripheral nerve regeneration in the rat model, its role in nerve regeneration of the central nervous systems of larger animals (e.g., rabbits, dogs, and goats) should be verified in the future. Overall, this study may provide an important experimental reference for the development of artificial implants for peripheral nerve regeneration.

## 4 Experimental Section

### Materials and Reagents

Sodium hyaluronate (HA, MW: 22 × 10^5^ Da) was purchased from the Nantong Feiyu Biotechnology Company. Cysteamine dihydrochloride (Cys) and 1‐(3‐Dimethylaminopropyl)−3‐ethylcarbodiimide hydrochloride (EDC) were purchased from Shanghai Aladdin Biochemical Technology Co., Ltd., and Shanghai Dubai, respectively. Pyrro‐1‐propionic acid was purchased from Shanghai Macklin Biochemical Co. Ltd. N‐hydroxysuccinimide (NHS) was purchased from Ron's reagent.

### Fabrication of the Self‐Healing Conductive Hydrogel

Briefly, 1.00 g of HA was dissolved in 450 mL of PBS solution at pH 5 and then poured into a solution of 0.956 g of EDC·HCl and 0.574 g NHS (HA:EDC·HCl:NHS = 1:2:2) to activate the carboxyl group of HA for 15 min. After the activation of the carboxyl group, 0.842 g of Cys was added to the HA solution and stirred in an ice bath for 12 h in the dark. The product was dialyzed for 3 days. Then, 0.521 g Py‐COOH was completely dissolved in PBS solution at pH 5, and after activation for 15 min through the addition of 1.434 g EDS, it was added to the above solution, which had been dialyzed for an additional 3 days, and stirred in an ice bath for 12 h. The product was on dialysis for an additional 3 days. The reaction product was lyophilized and stored until use. Then, a 0.05 g sample was weighed and dissolved in 1 mL deionized water, and stirred for 5 h, resulting in self‐formed glue. Then, 0.3 m FeCl_3_ solution was added, polymerized for 30 min, and left overnight at 4 °C. The excess ferric chloride solution was removed by cleaning.

### SEM Observation

To further confirm the porous structure of the hydrogel, freeze‐dried hydrogel samples were observed using scanning electron microscopy (ZEISS Gemini SEM 300) after gold sputtering.

### Characterization of HA‐Cys‐Py

The chemical structure of the amide bond was verified through ^1^H‐NMR spectroscopy (Bruker AVANCE III 400 MHz, Switzerland). HA, Cys, Py‐COOH, and freeze‐dried HA‐Cys and HA‐Cys‐Py were dissolved in D_2_O.

### Rheological Measurement and Mechanical Test

The storage modulus (*G*′) and loss modulus (*G*") of the hydrogels were evaluated using a Haake MARS rotational rheometer (Thermo Fisher Scientific, Waltham, MA, USA). After determining the maximum stress of the hydrogel, its self‐healing ability was evaluated. The oscillating strain oscillated between the small strain (γ = 1.0%) and large strain (γ_HA_ = 600%, γ_HASPy_ = 400%), with a duration of 120 and 300 s, respectively. The tensile measurements were performed at a constant rate of 10 mm min^−1^ using a universal tester. All the samples were cut into cuboids with a 10 mm length, 5 mm width, and 3 mm thickness. The typical tensile strength and fracture strain were obtained from the tensile stress–strain curves. Each group contained three parallel samples. The self‐healing hydrogels were subjected to the same tensile tests.

### Replacement of Bullfrog Sciatic Nerves with HASPy Hydrogel Ex Vivo

Positive and negative stimulation electrodes were connected to the proximal and distal sciatic nerves of the bullfrogs, respectively. In the experiment, the nerve and gastrocnemius muscles of the bullfrogs were continuously soaked in Ren's solution to maintain their physiological activity. The sciatic nerve defect was replaced by either HA or self‐repairing hydrogels. Electromyography was performed using a bl‐420S biological experimental system.

### Cell Cultures

L929 or RSC 96 Schwann cells (National Collection of Authenticated Cell Cultures, China) were cultured in a 5% carbon dioxide humidified incubator in Dulbecco's modified Eagle's medium (DMEM) supplemented with 10% fetal bovine serum and 1% penicillin/streptomycin. The culture medium was updated every 2 days.

### Cell Cytotoxicity Assay

L929 or RSC 96 Schwann cells were removed, washed with PBS buffer three times, and digested with 3 mL trypsin for 3 min. After the digestion was terminated, centrifugation and suspension were performed for counting.

The cells were inoculated on the hydrogel at a density of 1 × 10^4^ cells per well with a total volume of 100 µL in each well of a 24‐well plate. The cells were cultured in a CO_2_ incubator at 37 °C for 1 or 3 days. The original medium was discarded, the cells were washed with PBS buffer, and 10% CCK‐8 was added. After 1–2 h of incubation, 100 µL of supernatant per well was transferred to a new 96‐well plate, and the absorbance was measured at 450 nm using a microplate reader. Tests were performed on three independently prepared samples, and the results were expressed as the mean ± SD.

Cell viability was measured using live/dead staining. In addition, 250 µL Calcein‐CM/PI living and dead cell dye diluted 1000 times were added to each well and incubated at 37 °C for 30 min away from light. Finally, fluorescence was observed and photographed using an inverted microscope.

### High‐Throughput RNA‐seq

For transcriptomic sequencing, Schwann cells were seeded onto the HASPy hydrogel and TCP in a 6‐well culture plate for 3 days. Schwann cells that had grown to a certain density were collected, and TRIzol was used for total RNA isolation. The isolated total RNA was stored in liquid nitrogen ready for use. RNA concentration was determined and a total RNA quality inspection was performed. RNA libraries were then prepared by PCR amplification. Finally, sequencing was performed on the RNA‐seq platform in accordance with the manufacturer's protocol, and the data obtained were processed by Genewiz Co. Ltd. RNA‐seq data will be publicly available on the database of the National Genomic Data Center (https://ngdc.cncb.ac.cn/) on 3rd November, 2024 (BioProject No. PRJCA012957).

### Enzyme‐Linked Immunosorbent Assay

The concentration of interleukin‐17 (IL‐17) was measured in the supernatant collected after Schwann cells were incubated with the HASPy hydrogel for 3 days. An ELISA kit (immunoassay, China) was used in accordance with the manufacturer's instructions and was measured using a microplate reader at a wavelength of 450 nm.

### Inhibition of IL‐17 RA

The IL‐17 RA inhibitor ixekizumab (10 µg mL^−1^, GlpBio) was used to verify the possible interaction between CD44 and IL‐17RA; the results were then analyzed with immunofluorescence staining. Afterward, 100 µL hydrogel was placed in a 24‐well plate overnight during UV irradiation, and Schwann cells were seeded on the hydrogel at a density of 1 × 10^5^ cells per well. TCP was used as a control. Cells were treated with 1 mL DMEM per well and 10 µg mL^−1^ ixekizumab, whereas cells without inhibitors were used as controls. Schwann cells were co‐cultured for 3 days and immunostained with anti‐IL‐17RA receptor antibody (Abcam, USA).

### RT‐qPCR

RT‐qPCR was used to detect the expression levels of different genes in Schwann cells on the hydrogel. Cells were first cultured on the hydrogel for three days, and RNA from the Schwann cells was lysed using TRIzol (Gibco, USA), with the TCP group as the control. Reverse transcription of cDNA was performed using a reverse transcription kit. Finally, a real‐time PCR test was performed using a real‐time 2 × SYBR Green PCR mix kit (Solarbio, China) in accordance with the manufacturer's instructions. The primer sequences for MBP, NGF, *S100‐β*, and IL‐17RA were as follows: rat *Mbp* (forward, 5′‐ACACGGGCATCCTTGACTC‐3′: reverse, 5′‐GGTCCTCTGCGACTTCTGG‐3′), rat *Ngf* (forward, 5′‐GCTGGACCCAAGCTCAC‐3′; reverse, 5′‐CCCTCTGGGACATTGCTATC‐3′), rat glyceraldehyde‐3‐phosphate dehydrogenase (forward, 5′‐AACGACCCCTTCATTGAC‐3′; reverse, 5′‐TCCACGACATACTCAGCAC‐3′), rat *S100b* (forward, 5′‐ACTGAGGGACGAAATCAACA‐3′; reverse, 5′‐ CAACGGAGGTGCTATTGGTA‐3′), rat *Il17ra* (forward, 5′‐ACCCAAACCACAAATCCAAG‐3′; reverse, 5′‐ TGTGTCCAAGGTCTCCACAG‐3′), rat *Il17* (forward, 5′‐CACTCCTTCCGGCTAGAGAA‐3′; reverse, 5′‐CACATGGCGAACAATAGGG‐3′), rat *Cd44* (forward, 5′‐GCATTGCAGTCAACAGTC‐3′; reverse, 5′‐CCTTGTTCACCAAATGCACCA‐3′).

### Molecular Dynamic (MD) Simulations

Discovery Studio software was used to carry out the docking of the HASPy micromolecule onto the IL‐17A compound protein to explore the critical residues and interaction energies of the complexes. The IL‐17A compound protein (receptor) was obtained from RCSB PDB (5HHV.pdb). An HASPy micromolecule (ligand) was constructed and optimized. The ZDOCK model block was performed for IL‐17A and HASPy interactions. To investigate the mechanism of interaction, MD simulations were performed for each docked complex to monitor the stability of all backbone atoms of IL‐17A and HASPy micromolecules.

### Surgical Procedure

All procedures associated with the animal studies were approved by the Animal Ethics Committee of Nantong University (Approval No. S20200314‐041, China). Twenty‐four healthy female Sprague Dawley (SD) rats (age: 8 weeks, 200–250 g) were randomly divided into three groups: HASPy hydrogel, HA hydrogel, and injury. During surgery, all rats were anesthetized with a chloral hydrate sodium pentobarbital mixture (0.3 mL per 100 g body weight), the fur of the right femur was clipped, and the area was sterilized with iodine. The skin and muscles were then cut open to expose the sciatic nerve in the right hind leg. All animals were subjected to a crush injury using hemostatic forceps, 5 mm proximal to the bifurcation of the sciatic nerve. The first sound of the forceps compressed the nerve once every 5 s. When the forceps were reopened, the entire crush area was translucent. Then, 20 µL of hydrogel was injected into the injured site using a 1 mL syringe. The rats in the injury group were left untreated. The muscles and skin were closed using 4‐0 surgical sutures. All rats were allowed to recover, and all changes were monitored regularly. Animals were euthanized at a specific time during anesthesia. The nerves were collected and histologically examined.

### Tissue Processing and H&E Histological Evaluations

Thirty days after surgery, 3–5 SD rats from each group were randomly selected and euthanized. Sciatic nerves (*n* = 3 in each group) were fixed with 4% paraformaldehyde at 4 °C for 24 h and dehydrated with 30% sucrose solution for 48 h. The nerve tissue was embedded with an optimal cutting temperature (OCT) compound and then frozen. It was then cut into 5 µm slices either transversely or lengthwise using a frozen slicer. The slices were randomly divided into two groups: one group was stained for immunofluorescence and the other group with H&E. The sections were stained using a standard H&E protocol. The hematoxylin dye solution was applied for 5–10 min, and the excess dye was washed off with distilled water and differentiated for 2–30 s. After washing with water for 10 min, the eosin dye solution was applied for 30 s–2 min, alcohol gradient dehydrated for 10 s, placed in xylene transparent for 10 min, sealed and dried, and observed under a microscope.

### Immunostaining and Imaging

Sections or cells for immunostaining were washed with PBS three times, infiltrated with 1%Triton‐X 100 at 4 °C for 40 min, sealed with 5% BSA for 1 h, and then oscillated overnight with mouse anti‐*S100‐β* antibodies and rabbit anti‐Tuj1 antibodies at 4 °C. On day 2, after rewarming at room temperature for 1 h and rinsing with PBST three times, goat anti‐mouse (IgG H+L, Alexa Fluor 488, Abcam) and goat anti‐rabbit (IgG H+L, Alexa Fluor 647, Abcam) secondary antibodies were incubated in the dark at room temperature for 2 h. The sections were washed once with PBST, stained with DAPI, and incubated at room temperature for 30 min in the dark. The dye solution was discarded, and the cells were observed under an inverted fluorescence microscope.

### Gastrocnemius Muscle Evaluation

Thirty days after surgery, four SD rats from each group were selected for observation of the electrophysiological response, wet weight ratio, and gastrocnemius muscle fiber area. Motor function was assessed using an electrophysiological data acquisition system. The sciatic nerve on the injured side was carefully exposed to anesthesia in rats. Three electrodes were used to provide neural electrical stimulation, and the corresponding amplitudes were recorded using the acquisition system. The gastrocnemius muscles of the injured and contralateral hind limbs were carefully dissected and weighed to calculate the wet‐to‐weight ratio. The gastrocnemius muscle was then fixed with 4% paraformaldehyde at 4 °C for 24 h. The muscle was embedded in OCT to obtain frozen sections (thickness, 5 µm) for H&E staining. The stained sections were observed under a light microscope. Eight images were obtained from each sample, and the area of the gastrocnemius fibers was quantified using the Image software.

### Walking Track Analysis

Every 5 days after hydrogel implantation, the rats—the hind paws of which were both stained with black ink—were allowed to walk on a narrow track covered with a white sheet of paper. Three animals per group were selected, each rat was made to perform three repetitions of the exercise, and the parameters were averaged. Footprints of the rats are shown in Figure 5A. Combined with the analysis of the sciatic functional index (SFI), the calculation formula was as follows: Sciatic Functional Index = −38.3 (*L*_Exp_–*L*_Ctrl_)/(*L*_Ctrl_) + 109.5 (*M*_Exp_–*M*_Ctrl_)/(*M*_Ctrl_) + 13.3 (*S*_Exp_–*S*_Ctrl_)/(*S*_Ctrl_) − 8.8, where *M* is the distance between the first and fifth toes, *L* is the length of the paw, *S* is the distance between the second and fourth toes, Exp is the experimental paw, and Ctrl is the normal paw. The measurements were performed by an investigator blinded to the experimental group.

### TEM Investigation of Myelinated Nerve Fibers

Regenerated nerves were collected 30 days post‐operation, fixed with 2.5% glutaraldehyde for 3 h and subsequently in 1% osmium tetroxide solution for 1 h, washed, dehydrated, and embedded in epoxy resin. Ultrathin sections (70 nm) were prepared for TEM analysis. Ten images were obtained for each sample. The average g‐ratio (based on area), average diameter of myelinated nerve fibers, and average thickness of the myelin sheath were quantified using the Image software.

### Statistical Analysis

All data are expressed as the mean ± SD. Student's *t*‐test or one‐way analysis of variance (ANOVA) was used to statistically analyze the data with the Origin 8.0 software. Statistical significance was set at *p* < 0.05.

## Conflict of Interest

The authors declare no conflict of interest.

## Author Contributions

H.X., S.W., and Y.J. contributed equally to this work. H.X., B.L., Y.Y., and H.Y. performed conceptualization. S.W., B.L., and Y.X. performed methodology. S.W., Y.J., S.W., and F.X. performed investigation. H.X., S.W., and H.Y. performed visualization. B.L., Y.Y., and H.Y. performed supervision. H.X., S.W., Y.J., and H.Y. wrote the original draft. H.X., S.W., and H.Y. wrote, reviewed, and edited the manuscript.

## Supporting information

Supporting Information

## Data Availability

The data that support the findings of this study are available from the corresponding author upon reasonable request.

## References

[1] G. Li , T. Zheng , L. Wu , Q. Han , Y. Lei , L. Xue , L. Zhang , X. Gu , Y. Yang , Sci. Adv. 2021, 7, eabi5812. 34233882 10.1126/sciadv.abi5812 PMC8262819

[2] P. Deng , F. Chen , H. Zhang , Y. Chen , J. Zhou , Adv. Healthcare Mater. 2022, 11, 2200115. 10.1002/adhm.202200115 35396930

[3] J. Xu , C.‐W. Wong , S.‐h. Hsu , Chem. Mater. 2020, 32, 10407.

[4] a) R. Balint , N. J. Cassidy , S. H. Cartmell , Acta Biomater. 2014, 10, 2341; 24556448 10.1016/j.actbio.2014.02.015

[5] B. Bideau , L. Cherpozat , E. Loranger , C. Daneault , Ind. Crops Prod. 2016, 93, 136.

[6] D. A. Gyles , L. D. Castro , J. O. C. Silva Jr. , R. M. Ribeiro‐Costa , Eur. Polym. J. 2017, 88, 373.

[7] D. L. Gan , L. Han , M. H. Wang , W. S. Xing , T. Xu , H. P. Zhang , K. F. Wang , L. M. Fang , X. Lu , ACS Appl. Mater. Interfaces 2018, 10, 36218. 30251533 10.1021/acsami.8b10280

[8] S. Talebian , M. Mehrali , N. Taebnia , C. P. Pennisi , F. B. Kadumudi , J. Foroughi , M. Hasany , M. Nikkhah , M. Akbari , G. Orive , A. Dolatshahi‐Pirouz , Adv. Sci. 2019, 6, 1801664. 10.1002/advs.201801664 PMC6702654 31453048

[9] G. Vilarino‐Feltrer , C. Martinez‐Ramos , A. Monleon‐de‐la‐Fuente , A. Valles‐Lluch , D. Moratal , J. A. Barcia Albacar , M. Monleon Pradas , Acta Biomater. 2016, 30, 199. 26518102 10.1016/j.actbio.2015.10.040

[10] a) J. H. Collier , J. P. Camp , T. W. Hudson , C. E. Schmidt , J Biomed. Mater. Res. 2000, 50, 574; 10756316 10.1002/(sici)1097-4636(20000615)50:4<574::aid-jbm13>3.0.co;2-i

[11] X. Song , H. Gong , S. Yin , L. Cheng , C. Wang , Z. Li , Y. Li , X. Wang , G. Liu , Z. Liu , Adv. Funct. Mater. 2014, 24, 1194.

[12] D. Das , T. T. H. Pham , I. Noh , Colloid Surf. B 2018, 170, 64. 10.1016/j.colsurfb.2018.05.059 29879635

[13] R. Yang , G. Li , C. Zhuang , P. Yu , T. Ye , Y. Zhang , P. Shang , J. Huang , M. Cai , L. Wang , W. Cui , L. Deng , Sci. Adv. 2021, 7, eabg3816. 34162547 10.1126/sciadv.abg3816 PMC8221628

[14] J. Park , J. Jeon , B. Kim , M. S. Lee , S. Park , J. Lim , J. Yi , H. Lee , H. S. Yang , J. Y. Lee , Adv. Funct. Mater. 2020, 30, 2003759.

[15] a) Z. Zheng , Z. Guo , F. Zhong , B. Wang , L. Liu , W. Ma , C.‐y. Yu , H. Wei , J. Control. Release 2022, 347, 127; 35460706 10.1016/j.jconrel.2022.04.010

[16] C. Liu , F. Li , G. Li , P. Li , A. Hu , Z. Cui , Z. Cong , J. Niu , ACS Appl. Mater. Interfaces 2022, 14, 9608. 35143174 10.1021/acsami.1c23810

[17] M. Dong , B. Shi , D. Liu , J.‐H. Liu , D. Zhao , Z.‐H. Yu , X.‐Q. Shen , J.‐M. Gan , B.‐L. Shi , Y. Qiu , C.‐C. Wang , Z.‐Z. Zhu , Q.‐D. Shen , ACS Nano 2020, 14, 16565. 33025785 10.1021/acsnano.0c05197

[18] J. M. Shao , B. S. Zhou , A. J. Di Bilio , L. J. Zhu , T. L. Wang , C. Qi , J. Shih , Y. Yen , Mol. Cancer Ther. 2006, 5, 586. 16546972 10.1158/1535-7163.MCT-05-0384

[19] F. Meng , W. E. Hennink , Z. Zhong , Biomaterials 2009, 30, 2180. 19200596 10.1016/j.biomaterials.2009.01.026

[20] Z. Li , L. Liu , Y. Chen , Acta Biomater. 2020, 110, 119. 32438111 10.1016/j.actbio.2020.04.034

[21] a) L. Wang , B. Li , F. Xu , Y. Li , Z. Xu , D. Wei , Y. Feng , Y. Wang , D. Jia , Y. Zhou , Biomaterials 2017, 145, 192; 28869865 10.1016/j.biomaterials.2017.08.039

[22] a) P. Khoshakhlagh , M. J. Moore , Acta Biomater. 2015, 16, 23; 25617804 10.1016/j.actbio.2015.01.014

[23] A. Nirula , J. Nilsen , P. Klekotka , G. Kricorian , N. Erondu , J. E. Towne , C. B. Russell , D. A. Martin , A. L. Budelsky , Rheumatology 2016, 55, ii43. 27856660 10.1093/rheumatology/kew346

[24] J. J. Yu , S. L. Gaffen , Front. Biosci.‐Landmark 2008, 13, 170. 10.2741/2667 17981535

[25] H. Nisar , U. Pasha , M. U. Mirza , R. Abid , K. Hanif , H. N. Kadarmideen , S. Sadaf , Immunol Invest 2021, 50, 416. 32543936 10.1080/08820139.2020.1775642

[26] T. L. Lopez‐Silva , C. D. Cristobal , C. S. E. Lai , V. Leyva‐Aranda , H. K. Lee , J. D. Hartgerink , Biomaterials 2021, 265, 120401. 33002786 10.1016/j.biomaterials.2020.120401 PMC7669633

[27] Y. Li , S. Lv , H. Yuan , G. Ye , W. Mu , Y. Fu , X. Zhang , Z. Feng , Y. He , W. Chen , Adv. Funct. Mater. 2021, 31, 2010215.

[28] A. Sliow , Z. Ma , G. Gargiulo , D. Mahns , D. Mawad , P. Breen , M. Stoodley , J. Houang , R. Kuchel , G. C. Tettamanzi , R. D. Tilley , S. J. Frost , J. Morley , L. Longo , A. Lauto , Adv. Sci. 2019, 6, 1801212. 10.1002/advs.201801212 PMC6548953 31179205

[29] S. Naskar , V. Kumaran , Y. S. Markandeya , B. Mehta , B. Basu , Biomaterials 2020, 226, 119522. 31669894 10.1016/j.biomaterials.2019.119522

[30] H. Jiang , X. Wang , X. Li , Y. Jin , Z. Yan , X. Yao , W.‐E. Yuan , Y. Qian , Y. Ouyang , Mater Today Bio 2022, 13, 100211. 10.1016/j.mtbio.2022.100211 PMC8841887 35198959

[31] A. Thibodeau , T. Galbraith , C. M. Fauvel , H. T. Khuong , F. Berthod , Biomaterials 2022, 280, 121269. 34847434 10.1016/j.biomaterials.2021.121269

[32] L. Wang , C. Lu , S. Yang , P. Sun , Y. Wang , Y. Guan , S. Liu , D. Cheng , H. Meng , Q. Wang , J. He , H. Hou , H. Li , W. Lu , Y. Zhao , J. Wang , Y. Zhu , Y. Li , D. Luo , T. Li , H. Chen , S. Wang , X. Sheng , W. Xiong , X. Wang , J. Peng , L. Yin , Sci. Adv. 2020, 6, eabc6686. 33310851 10.1126/sciadv.abc6686 PMC7732202

[33] F. Rao , Y. Wang , D. Zhang , C. Lu , Z. Cao , J. Sui , M. Wu , Y. Zhang , W. Pi , B. Wang , Y. Kou , X. Wang , P. Zhang , B. Jiang , Theranostics 2020, 10, 1590. 32042324 10.7150/thno.36272 PMC6993237

[34] D. Hua , S. Gao , M. Zhang , W. Ma , C. Huang , Carbohydr. Polym. 2020, 247, 116743. 32829862 10.1016/j.carbpol.2020.116743

[35] a) J. Park , J. Jin , B. Kim , S. L. Min , J. Y. J. A. F. M. Lee , Adv. Funct. Mater. 2020, 30, 2003759;

[36] Y. Zhao , Y. Liang , S. Ding , K. Zhang , H.‐q. Mao , Y. Yang , Biomaterials 2020, 255, 120164. 32554132 10.1016/j.biomaterials.2020.120164

[37] S. Ozaki , E. Ebisui , K. Hamada , A. Z. Suzuki , A. Terauchi , K. Mikoshiba , Bioorg. Med. Chem. Lett. 2011, 21, 377. 21134746 10.1016/j.bmcl.2010.10.136

[38] D. L. Taylor , M. I. H. Panhuis , Adv. Mater. 2016, 28, 9060. 27488822 10.1002/adma.201601613

[39] M. Stettner , B. Lohmann , K. Wolffram , J.‐P. Weinberger , T. Dehmel , H.‐P. Hartung , A. K. Mausberg , B. C. Kieseier , J. Neuroinflammation 2014, 11, 63. 24678820 10.1186/1742-2094-11-63 PMC3977670

[40] D. Vigetti , E. Karousou , M. Viola , S. Deleonibus , G. De Luca , A. Passi , Biochim. Biophys. Acta, Gen. Subj. 2014, 1840, 2452. 10.1016/j.bbagen.2014.02.001 24513306

[41] a) H. Huang , H. Liu , R. Yan , M. Hu , Neurochem. Res. 2017, 42, 3515; 28993995 10.1007/s11064-017-2399-1

[42] A. Ouhtit , B. Rizeq , H. A. Saleh , M. D. M. Rahman , H. Zayed , Int. J. Biol. Sci. 2018, 14, 1782. 30443182 10.7150/ijbs.23586 PMC6231220

[43] A. L. Herbert , M.‐m. Fu , C. M. Drerup , R. S. Gray , B. L. Harty , S. D. Ackerman , T. O'Reilly‐Pol , S. L. Johnson , A. V. Nechiporuk , B. A. Barres , K. R. Monk , P. Natl. Acad. Sci. USA 2017, 114, E9153. 10.1073/pnas.1711088114 PMC5664533 29073112

[44] C. W. Wu , X. Z. He , W. J. Weng , T. F. Zhang , D. H. Huang , K. Cheng , Z. B. Chen , Chem. Eng. J. 2022, 443, 136508.

