# Supplementary material for: A Bioinspired Self‐Healing Conductive Hydrogel Promoting Peripheral Nerve Regeneration
Source: Adv Sci (Weinh). 2023 Aug 23;10(28):2302519. doi: 10.1002/advs.202302519 (PMC10558694; doi:10.1002/advs.202302519)
Supplement: Supplementary file 1 — Supporting Information [file ADVS-10-2302519-s001.pdf]

ADVANCED SCIENCE
Open Access

Supporting Information

for *Adv. Sci.*, DOI 10.1002/advs.202302519

A Bioinspired Self-Healing Conductive Hydrogel Promoting Peripheral Nerve Regeneration

Hongyun Xuan, Shuyuan Wu, Yan Jin, Shuo Wei, Feng Xiong, Ye Xue, Biyun Li, Yumin Yang* and Huihua Yuan**

Supplementary Materials for

A bioinspired self-healing conductive hydrogel promoting peripheral nerve regeneration

Hongyun Xuan, Shuyuan Wu, Yan Jin, Shuo Wei, Feng Xiong, Ye Xue, Biyun Li*, Yumin Yang*, Huihua Yuan*

*Corresponding author. Email: libiyun1986@163.com, yangym@ntu.edu.cn, yuanhh@ntu.edu.cn

This PDF file includes:

Materials and Methods
Figs. S1 to S10
Tables S1 to S6

Supplementary Materials

Materials and Methods

FTIR characterization of HASPy hydrogel

The frequency range of the FTIR spectroscope was set to 4000–500 cm^{-1}, and each sample was scanned 32 times.

UV–Vis characterization of HASPy

UV–Vis absorbance spectra of HASPy films deposited on the glass slides were recorded using spectrometer in the 850–300 nm wavelength range.

Determination of graft ratio of HA-Cys-Py

Since HA-Cys-Py was synthesized based on amide bonds, the grafting ratio of HA-Cys-Py can be measured by the residual carboxyl group content. The content of carboxyl group (CC) was measured by conductivity titration method. The sample to be tested was weighed with 0.3g of dried HA-Cys-Py and added with 55mL distilled water and 5 mL0.01M sodium chloride solution. The sample was fully stirred with magnetic agitator (the concentration was about 0.5%), and then 0.1M HCl was added to adjust the pH value of the mixture 3.0. Magnetic stirring 1h to ensure the acidification of the base. Then, 0.04M NaOH solution was titrated at the rate of 0.1mL/min, and the conductivity meter was used to monitor the conductivity of the mixture during the reaction process. The titration was stopped until the pH value of the solution was 11, and the carboxyl group content was calculated according to the consumption of sodium hydroxide and the conductivity.

Draw the curve of sodium hydroxide consumption (volume, ml) *vs.* conductivity according to the method mentioned above (Fig.S3), and calculate the carboxyl group content according to the formula:

$CC = 0.04\ mol/L \times (V_B - V_A)\ mL/0.3g$ (1)

Where V_A and V_B are respectively the x-coordinate values corresponding to the intersection of the left and right tangent lines of the conductivity curve and the horizontal line of the lowest point of the curve.

$\text{Graft ratio} = (CC_{HA} - CC_{HA\text{-}Cys\text{-}Py}) / CC_{HA} \times 100$ (2)

Electroactivity measurement

The conductivity of the hydrogels was measured using four probes and an electrochemical workstation. In 0.1 mol/L PBS electrolyte solution, a traditional three-electrode system with Pt as the counter electrode, an Ag/AgCl electrode as the reference electrode, and a hydrogel sample as the working electrode was used for electroactivity analysis at room temperature using an electrochemical workstation (CHI660D, China). Based on cyclic voltammetry (CV) measurements, the potential range was -0.8–0.2 V and the scanning rate 100 mV/s. The conductivity of the hydrogels is calculated according to the following formulas:

$V = I \times R$ (3)

$\sigma = L/RS$ (4)

$R = \rho L/S$ (5)

Where σ ($S \cdot cm^{-1}$) is the desired conductivity, L (cm) is the distance between the reference electrode and the working electrode, S (cm^2) is the cross-sectional area of the measured hydrogel, R (Ω) is the ohmic resistance, and ρ ($\Omega \cdot cm$) is the resistivity.

All-Atom Molecular Dynamics simulations method

Materials Studio software was used to simulate the interactions between HASPy chains with water molecules and iron ions under the Compass force field. A simulation crystal Cell was constructed by Amophous Cell calculation. For HASPy chains with water molecules (HASPy-W), two HASPy chains and 300 water molecules were placed in the box. For HASPy chains with iron ions, two HASPy chains, 300 water molecules, 40 Fe^{3+} and 120 Cl^- ions were added in the box. The initial size of the simulation unit cell is 30.03 Å × 30.03 Å× 30.03 Å, with the lattice parameters of $\alpha = \beta = \gamma = 90°$. The models were equilibrated at 298 K for 25 ps in the NPT ensemble.

Self-healing experiment

The self-healing behavior of hydrogels was directly observed. The original sample was usually made into rectangular strips (10 × 5 × 3 mm), and then the hydrogel was cut into two equal parts from the middle. The cut surfaces were then placed in full contact and the hydrogel was wrapped in a petri dish for self-healing. After 30 min of self-healing, the hydrogel completely returned to its original state, and tensile and tensile tests were conducted to evaluate the tensile strength before and after self-healing.

In vitro degradation test

First, 10 mg of hydrogel was placed in 1 mL PBS, and 10 mM DTT was added for incubation at 37 °C. PBS without DTT was used as the control group. Then images were obtained at regular intervals to record the morphologies of the hydrogels.

In vivo biocompatibility and degradation test

The hydrogel was disinfected overnight under a UV lamp and the surgical instruments were sterilized by autoclaving. Nine adult female Sprague-Dawley (SD) rats (200–230 g) were randomly divided into three groups. After anesthesia, the rats were placed on the surgical plate, and 10-mm incisions were made on the backs of the SD rats and 30 mg of hydrogel implanted. Skin tissue samples were collected on the 7th 14th, and 28th postoperative days (PODs). Tissue samples were fixed in paraformaldehyde for 24 h and dehydrated in sucrose for 48 h. The sample was then embedded in OCT at the optimum cutting temperature and cut into slices that were 8 μm thick. Finally, in vivo biocompatibility and hydrogel degradation were assessed using H&E staining.

Hemolysis assay

Hemolysis testing of the samples was performed according to the ISO 10993-4 procedure. An anticoagulant agent (3 wv% sodium citrate + 0.1 wv % citrate + 2.5 wv % glucose) was added to the red blood cells from SD rats at a ratio of 7:1. Then 2 mL of whole blood was diluted to 4.5 ml, and a 10 mg sample was placed in 10 mL PBS, and 0.2 mL blood was added, and the solution was incubated at 37 °C for 1 h. Then, the mixed solution was centrifuged at 3000 rpm for 5 min. The absorbance at a wavelength of 545 nm was measured using a UV spectrometer, and the hemolysis rate was calculated as follows:

Hemolysis percentage= (absorbance experimental group − absorbance negative group) / (absorbance positive group − absorbance negative group) ×100% (6)

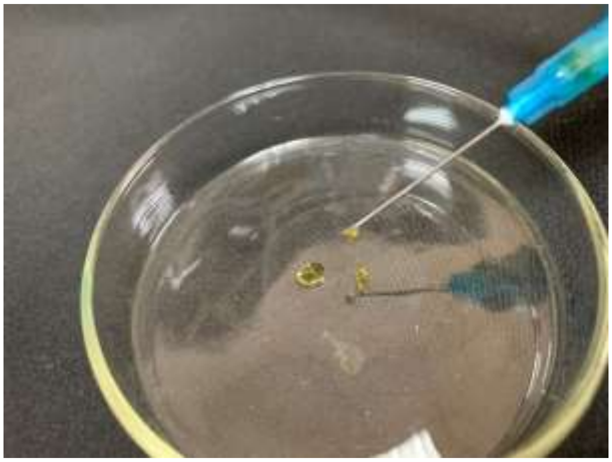

Fig.S1 Image of the HASPy hydrogel injected through the syringe with a needle

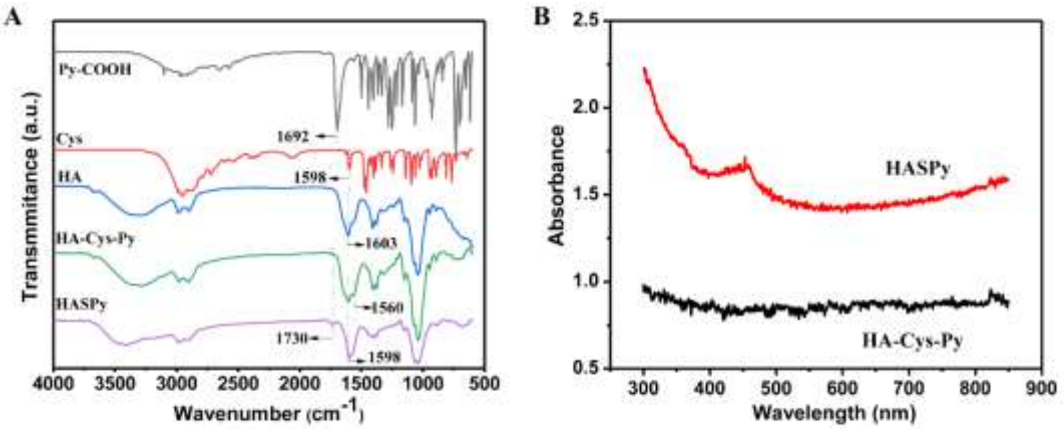

Fig.S2 (A) FTIR analysis of Py-COOH, Cys, HA, HA-Cys-Py and HASPy. (B) UV–Vis absorption spectroscopy of HA-Cys-Py and HASPy.

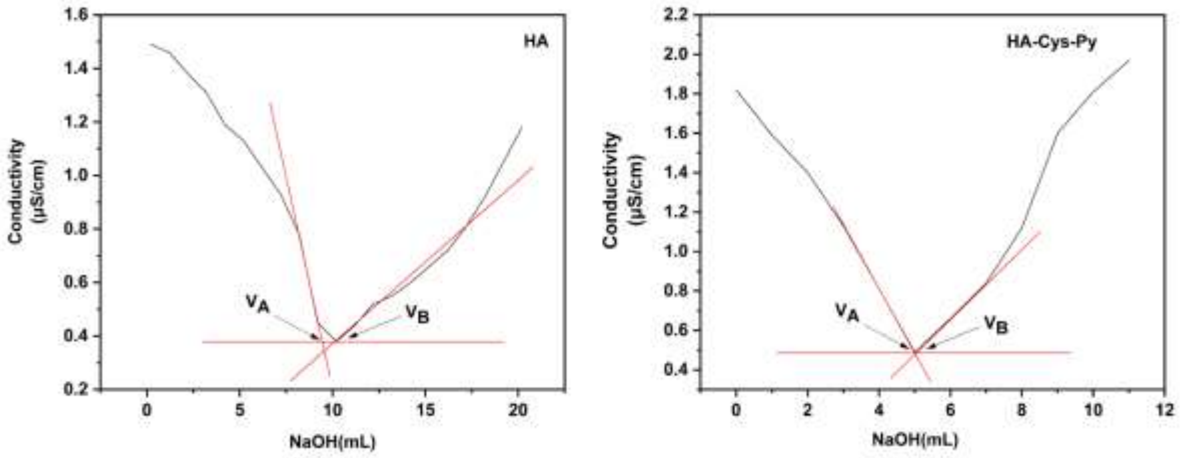

Fig. S3 The curve of sodium hydroxide consumption (volume, ml) vs. conductivity (left: HA, right: HA-Cys-Py)

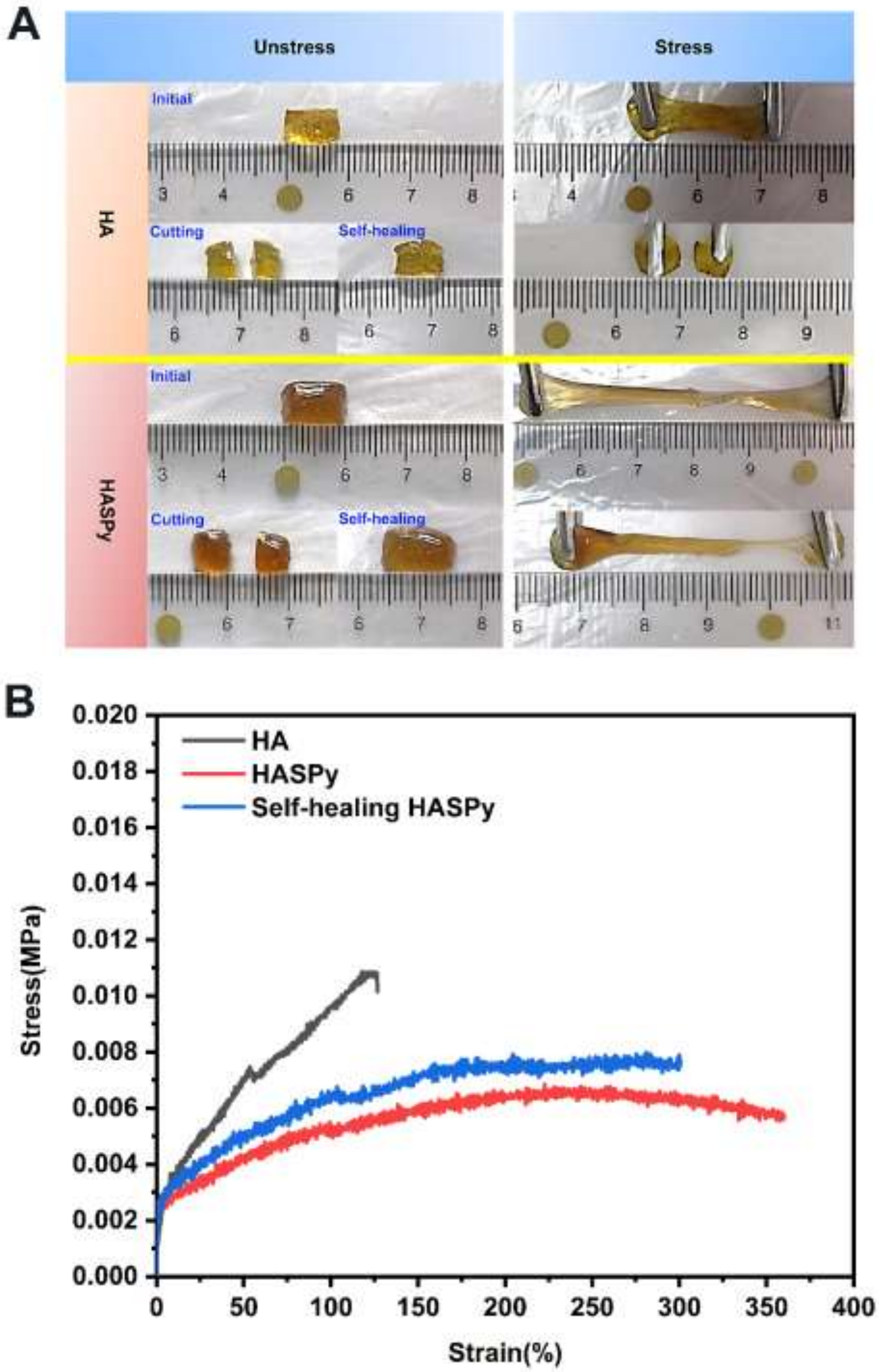

Fig. S4 Mechanical properties of hydrogels. (A) Images of free-standing HA hydrogel and HASPy hydrogel without stretching (left) and with stretching to elongation (right) before and after self-healing. (B) Stress changes with strain of HA hydrogels, before and after self-healing of HASPy hydrogels.

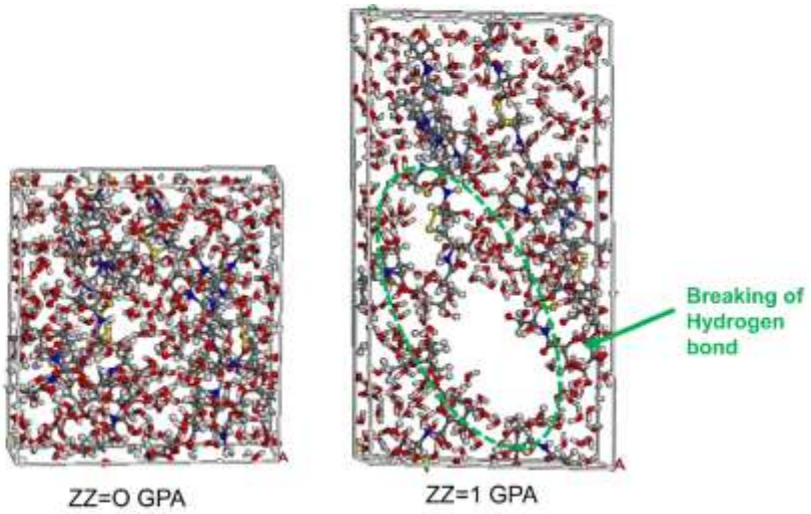

Fig. S5 Molecular dynamics simulations of HASPy-water.

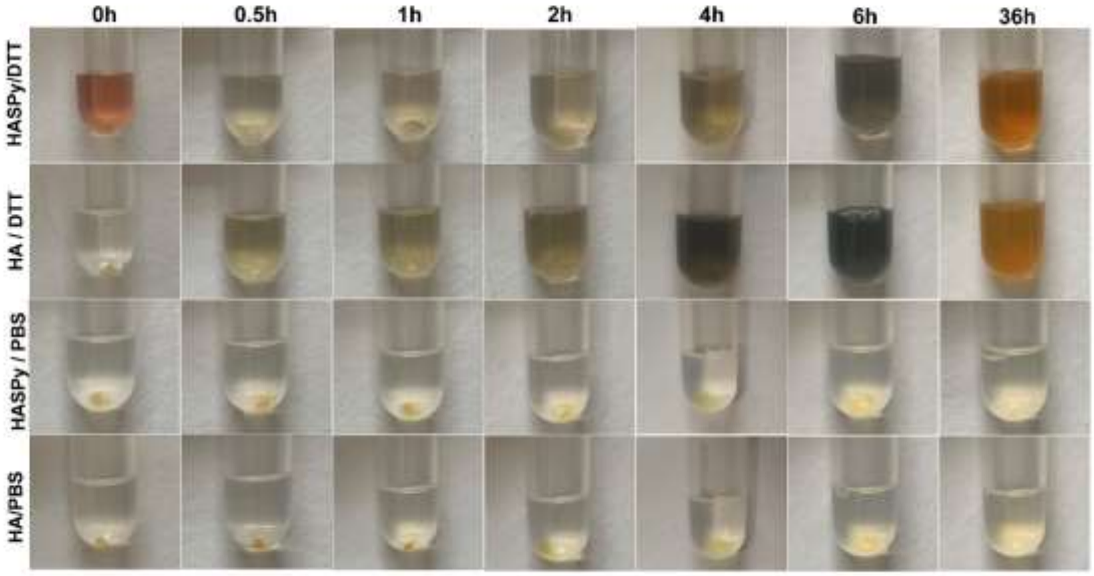

Fig. S6 Degradation behavior of hydrogel in vitro. Degradation image of HA and HASPy hydrogels over time from 0 to 36 h in DTT-plus PBS and PBS, respectively.

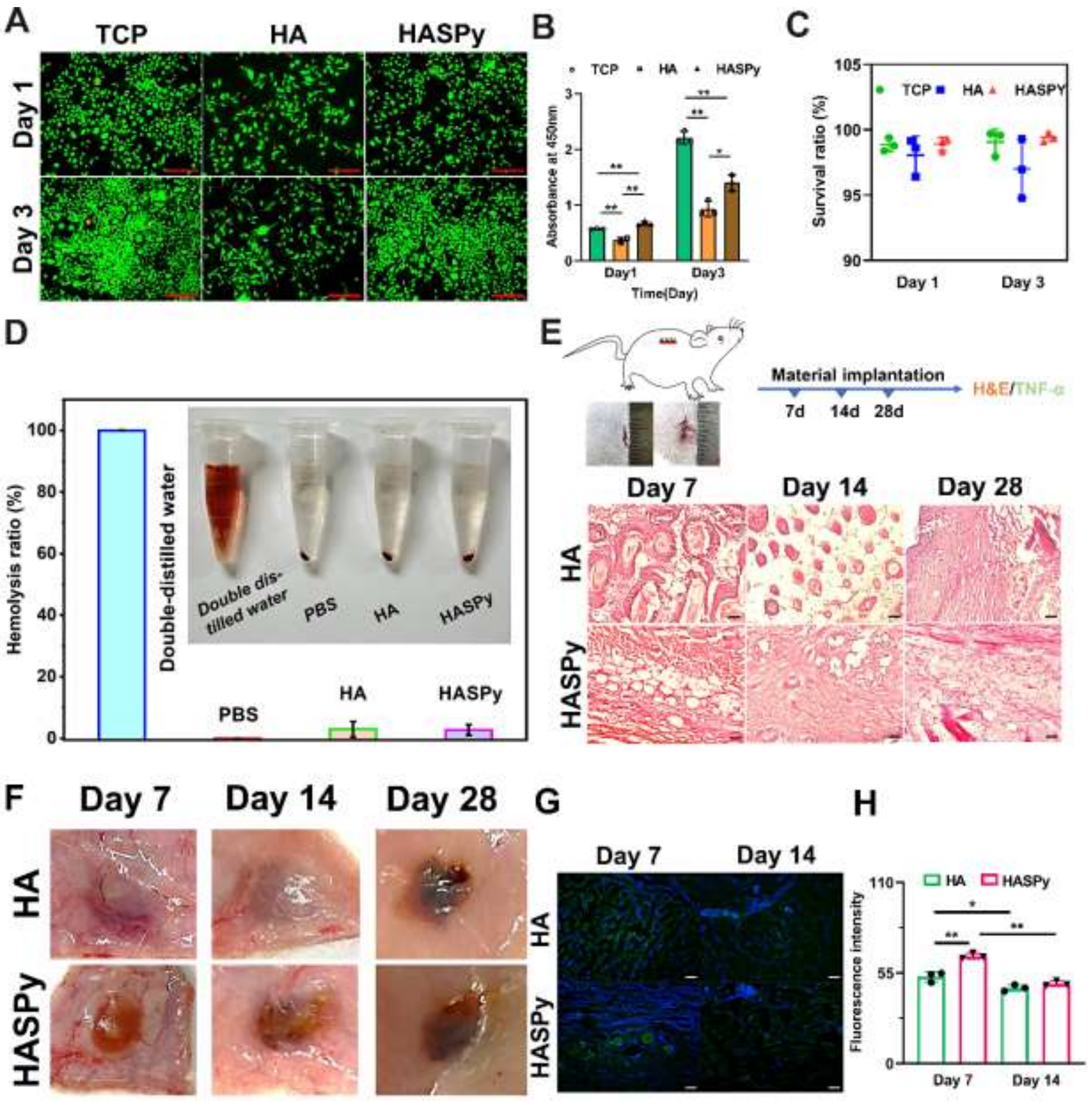

Fig. S7 In vitro and vivo biocompatibility of HASPy. (A) Live and dead cell staining of L929 cells. Scale bar: 100 μm. (B) The survival ratio. (C) Cell Counting Kit-8 (CCK-8) assay. (D) Hemolysis rate. (E) Subcutaneous biocompatibility of HA and HASPy hydrogels by H&E staining. Scale bar: 50 μm. (F) Image of subcutaneous biocompatibility of HA and HASPy hydrogels. (G) Immunofluorescence images of TNF-α after 7 days and 14 days of HA and HASPy hydrogels. Scale bar: 50 μm. (H) Fluorescence intensity of TNF-α.

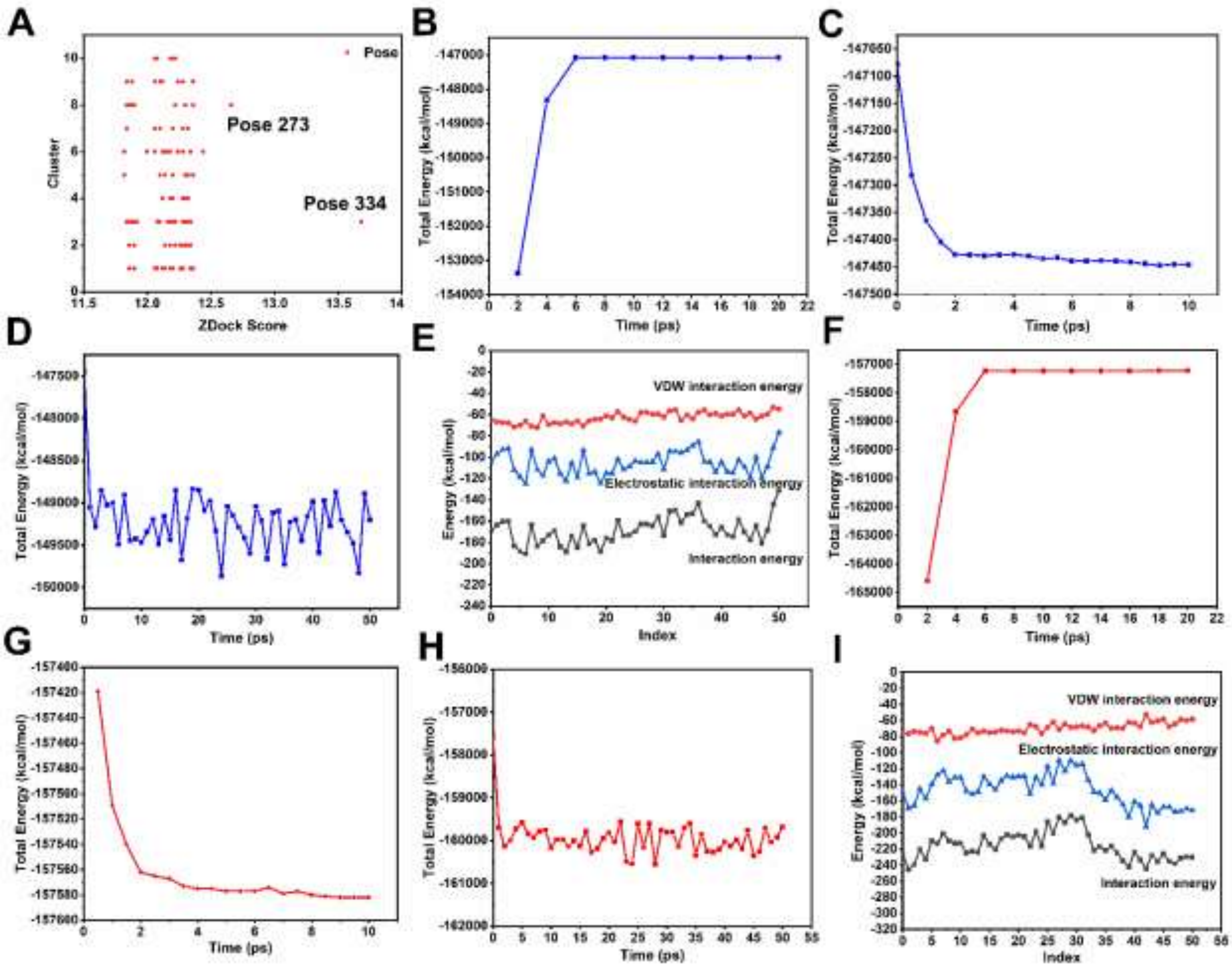

Fig. S8 (A) ZDock score of the clusters in ZDock simulation. (B-D) Time-total energy curves of the dynamic simulation (B) the healing/cooling simulation, (C) the equilibration simulation and (D) the production simulation). (E) The VDW interaction energy, electrostatic interaction energy and interaction energy curves of the all conformations in the last 50 ps of the production simulation. (F-H) Time-total energy curves of the dynamic simulation (F) the healing/cooling simulation, (G) the equilibration simulation and (H) the production simulation). (I) The VDW interaction energy, electrostatic interaction energy and interaction energy curves of the all conformations in the last 50 ps of the production simulation.

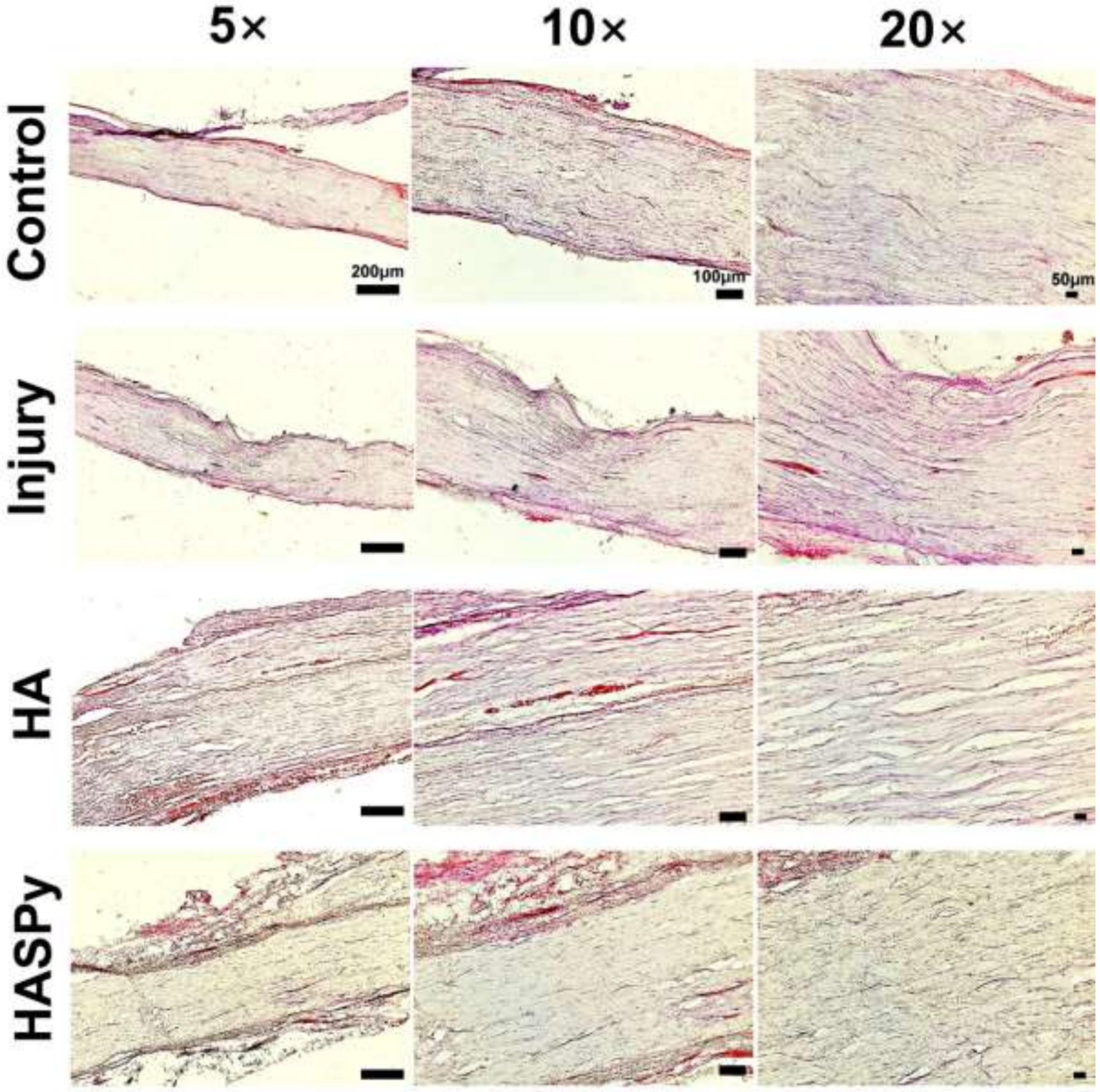

Fig. S9 Characterization of longitudinal section of sciatic nerve. H&E staining of the longitudinal section of the nerve.

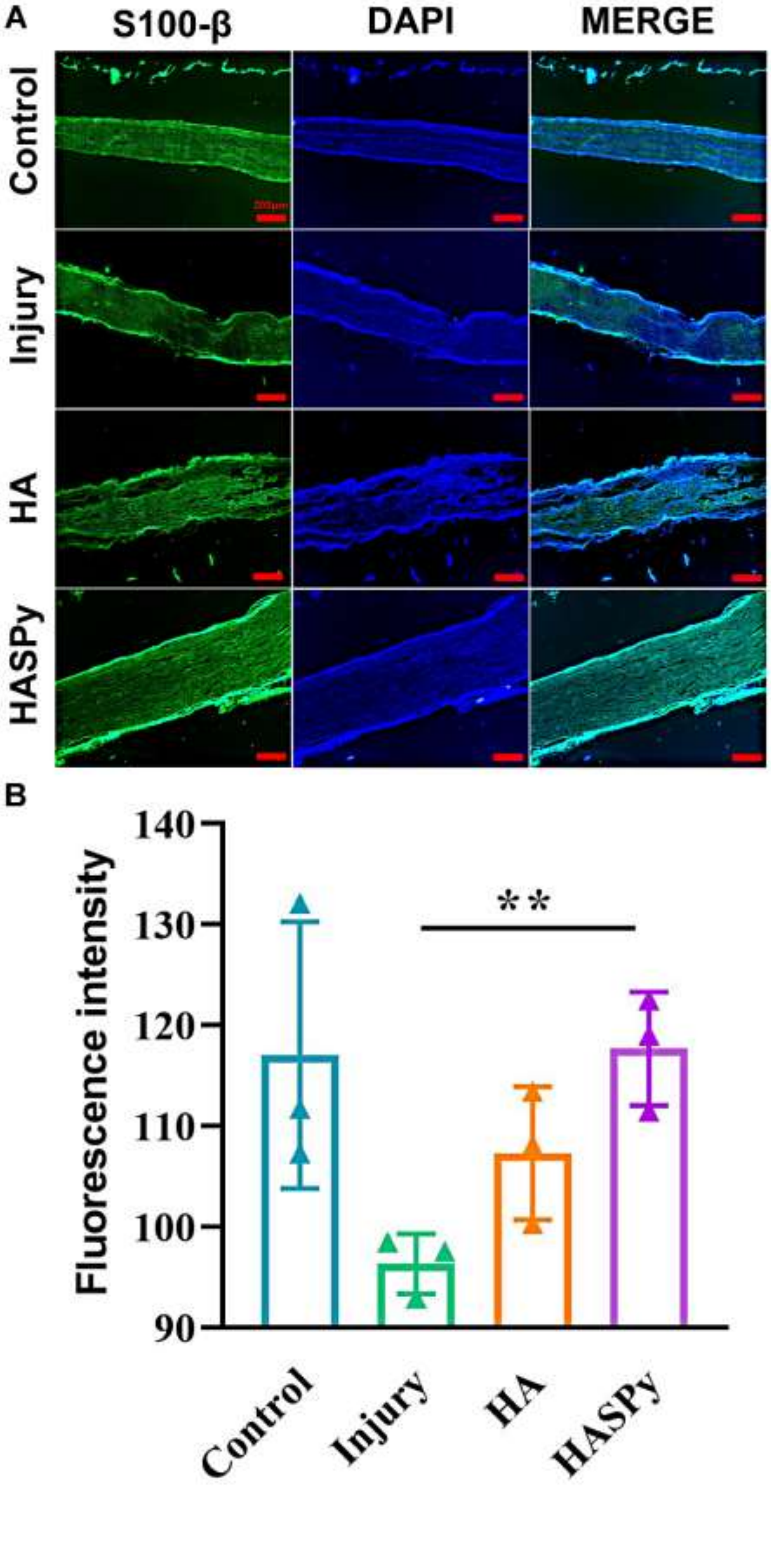
A
S100-β
DAPI
MERGE
Control
Injury
HA
HASPy
B
Fluorescence intensity
140
130
120
110
100
90
**
Control
Injury
HA
HASPy

Fig. S10 (A) Immunofluorescence staining image of the longitudinal section of a nerve (S100-β). (B) Statistics of fluorescence intensity of S100-β in control, injury, HA, HASPy. Scale bar: 200 μm.

TABLE

Table S1. The interaction energy (E_{int}), van der Waals energy (E_{vdw}) and electrostatic interaction energy (E_{ele}) between HASPy and IL-17A compound protein (IL-17(A, RAH)).

Conformation	E_{int} (kcal/mol)	E_{vdw} (kcal/mol)	E_{ele} (kcal/mol)
IL-17(A,RAH) /HASPy	-201.93	-72.09	-129.84

Table S2. The distance and angle DHY of the hydrogen bonds between HASPy and IL-17A compound protein (IL-17(A, RAH)).

Name	Distance	Angle DHY
A:GLN93:H_{E21} ··· O_{73}:HASPy	1.8	145.1
A:GLN93:H_{E22} ··· O_{133}:HASPy	2.1	161.5
HASPy:H_{181} ··· O:A:PRO91	3.0	104.4
HASPy:H_{182} ··· O:A:PRO91	2.2	138.7
H:SER30:H_{G} ··· O_{145}:HASPy	2.2	167.7
HASPy:H_{244} ··· O:H:SER30	2.4	151.8
HASPy:H_{178} ··· O:H:SER30	2.90	132.6
HASPy:H_{179} ··· O:H:SER30	2.48	143.2
HASPy:H_{245} ··· O:H:SER31	2.2	105.7
HASPy:H_{246} ··· O:H:SER31	2.7	161.9
H:SER31:H_{A} ··· O_{65}:HASPy	2.8	146.5
H:SER53:H_{G} ··· O_{73}:HASPy	2.0	134.7
H:SER53:H_{G} ··· O_{81}:HASPy	1.8	124.9
H:SER53:H_{B2} ··· O_{73}:HASPy	2.5	100.5
H:SER53:H_{B2} ··· O_{146}:HASPy	2.8	105.3
H:GLY5S:H_{N} ··· O_{146}:HASPy	2.1	132.9
H:SER74:H_{N} ··· O_{35}:HASPy	2.0	157.0
H:SER74:H_{G} ··· O_{35}:HASPy	1.8	152.2
H:SER74:H_{B1} ··· O_{35}:HASPy	3.0	90.6
H:LYS75:H_{E2} ··· O_{48}:HASPy	2.7	109.5
HASPy:H_{164} ··· O:H:ARG71	2.7	137.6
HASPy:H_{225} ··· O:H:SER53	2.6	132.6

Table S3. The interaction energy (Eint), van der Waals energy (Evdw), electrostatic interaction energy (Eele) and total hydrophohic interaction energy (Etotal)between HASPy and individual residues in IL-17(A, RAH).

Hydrophohic residue	E_{int} (kcal/mol)	E_{vdw} (kcal/mol)	E_{ele} (kcal/mol)
ILE66	-0.086995	-0.002611	-0.084384
ALA69	0.386252	-0.019751	0.406002
CYS71	0.98818	-0.030421	1.0186
LEU74	0.086842	-0.00506	0.091903
CYS76	-0.00105	-0.000706	-0.000344
MET87	-0.189936	-0.003526	-0.18641
VAL90	-2.194629	-1.748269	-0.44636
ILE92	-2.749146	-2.268302	-0.480844
ILE96	0.17449	-0.015125	0.189616
LEU97	-0.000387	-0.000049	-0.000339
VAL98	0.005142	-0.001682	0.006824
ILE115	-0.804715	-0.19755	-0.607165
LEU116	-5.910539	-3.640874	-2.269664
VAL117	0.997034	-0.197711	1.194745
VAL119	-1.04904	-0.062044	-0.986996
CYS121	-0.023291	-0.070416	0.047124
CYS123	-0.037994	-0.049974	0.01198
VAL124	-1.182498	-1.209063	0.026565
ILE127	-0.143883	-0.00485	-0.139033
VAL128	0.062285	-0.011031	0.073315
H_LEU-4	-0.02214	-0.000395	-0.021745
H_VAL2	-0.00567	-0.000003	-0.005667
H_LEU18	-0.000378	0	-0.000378
H_LEU20	0.105571	-0.00426	0.10983
H_CYS22	0.400647	-0.00374	0.404386
H_ALA23	0.054724	-0.006969	0.061692
H_ALA24	-0.18099	-0.003039	-0.177951
H_PHE27	-0.156423	-0.022285	-0.134138
H_PHE29	-0.483258	-0.507838	0.02458
H_ALA33	0.01608	-0.12316	0.13924
H_MET34	-0.158175	-0.016188	-0.141986
H_ALA50	0.10395	-0.000653	0.104603
H_ILE51	-1.27955	-0.272918	-1.006632
H_ILE69	0.055276	-0.029606	0.084882
H_LEU78	0.255156	-0.035527	0.290682
H_LEU80	-0.090309	-0.000958	-0.089351
H_LEU96	-0.078098	-0.070991	-0.007106
H_ILE97	-0.291816	-0.077977	-0.213839
H_VAL100	0.072341	-0.000258	0.072599
E_{total} (kcal/mol)		-13.35694	

Table S4. The interaction energy (Eint), van der Waals energy (Evdw) and electrostatic interaction energy (Eele) between HASPy and IL-17A compound protein (IL-17RA (H, L)).

Conformation	E_{int} (kcal/mol)	E_{vdw} (kcal/mol)	E_{ele} (kcal/mol)
IL-17RA(H,L)/HASPy	-245.69	-76.70	-168.99

Table S5. The distance and angle DHY of the hydrogen bonds between HASPy and IL-17A compound protein (IL-17RA (H, L)).

Name	Distance	Angle DHY
H:LYS43:H_{Z1} ··· O_{45}:HASPy	1.8	132.9
H:LYS43:H_{Z3} ··· O_{48}:HASPy	1.7	141.7
H:GLN1438:HE_{21} ··· O_{146}:HASPy	2.1	126.1
H:ALA168:H_{N} ··· O_{133}:HASPy	2.0	172.5
H:TYR176:H_{H} ··· O_{132}:HASPy	2.0	164.5
L:GLY41:H_{N} ··· O_{145}:HASPy	2.8	96.4
L:LYS103:H_{Z2} ··· O_{26}:HASPy	1.8	129.1
L:THR164:H_{G1} ··· O_{36}:HASPy	1.9	155.2
L:LYS167:H_{N} ··· O_{142}:HASPy	2.4	143.4
L:SER169:H_{G} ··· O_{57}:HASPy	2.4	118.6
HASPy:H_{236} ··· OD_{2}:L:ASP85	2.4	158.9
H:PRO167:H_{A} ··· O_{133}:HASPy	2.4	150.4
H:SER172:H_{B2} ··· O_{110}:HASPy	2.6	156.7
L:PRO40:H_{A} ··· O_{145}:HASPy	2.8	99.7
L:GLY101:H_{A2} ··· O_{22}:HASPy	2.3	128.8
L:TAR164:H_{B} ··· O_{49}:HASPy	2.6	116.9
HASPy:H_{156} ··· O:L:H1S8	2.5	160.7
HASPy:H_{157} ··· O:L:GLY100	2.6	118.0
HASPy:H_{178} ··· O:L:GLY41	2.6	132.78
HASPy:H_{179} ··· O:L:PRO40	2.5	145.4
HASPy:H_{180} ··· O:L:GLY41	2.4	149.0
HASPy:H_{214} ··· O:H:LEU170	3.0	110.0
HASPy:H_{230} ··· O:L:LYS167	2.6	171.1

Table S6. The interaction energy (Eint), van der Waals energy (Evdw), electrostatic interaction energy (Eele) and total hydrophohic interaction energy (Etotal)between HASPy and individual residues in IL-17RA (H, L).

Hydrophohic residue	E_{int} (kcal/mol)	E_{vdw} (kcal/mol)	E_{ele} (kcal/mol)
H_LEU4	0.322279	-0.000150	0.322429
H_LEU5	-0.089535	-0.000865	-0.088670
H_LEU11	0.005733	-0.000043	0.005776
H_VAL12	0.105233	-0.000034	0.105267
H_VAL37	0.000036	-0.000000	0.000037
H_ALA40	-0.618720	-0.445730	-0.172990
H_LEU45	0.084089	-0.043171	0.127260
H_LEU82	0.012339	-0.000001	0.012341
H_ALA84	-0.392329	-0.093272	-0.299057
H_ALA88	-0.142763	-0.076873	-0.065890
H_VAL89	-0.248065	-0.543941	0.295875
H_CYS92	-0.094211	-0.000067	-0.094144
H_LEU108	-1.464209	-0.464993	-0.999216
H_VAL109	0.522229	-0.018030	0.540259
H_VAL111	0.021125	-0.010758	0.031883
H_ALA114	-0.381407	-0.024865	-0.356542
H_LEU141	-0.014229	-0.000026	-0.014202
H_VAL142	0.861419	-0.002829	0.864248
H_PHE146	-0.933635	-0.071143	-0.862492
H_VAL150	0.899929	-0.046612	0.946541
H_VAL152	-0.360785	-0.001487	-0.359298
H_VAL163	0.189283	-0.021605	0.210889
H_PHE166	0.204845	-0.379100	0.583945
H_ALA168	-9.149320	-2.883360	-6.265960
H_VAL169	-2.983793	-2.191046	-0.792747
H_LEU170	-7.268606	-6.349358	-0.919248
H_LEU175	0.423986	-0.122182	0.546169
H_LEU178	-0.665667	-0.238022	-0.427645
H_VAL181	-0.236897	-0.000297	-0.236600
H_VAL198	-0.003046	-0.000002	-0.003044
L_MET3	-0.035902	-0.000871	-0.035031
L_LEU4	-0.120553	-0.004687	-0.115866
L_VAL11	-0.284520	-0.038315	-0.246205
L_ILE21	0.078218	-0.003817	0.082034
L_CYS23	-0.014124	-0.001470	-0.012653
L_VAL47	0.001559	-0.000001	0.001560
L_LEU73	-0.016360	-0.000031	-0.016329
L_ALA84	-0.186072	-0.097178	-0.088894
L_CYS88	-0.127517	-0.008006	-0.119511
L_PHE98	-0.007377	-0.004495	-0.002882
L_lEU104	0.484668	-0.081648	0.566316
L_VAL106	-0.264268	-0.000642	-0.263626
L_LEU107	0.002505	-0.000005	0.002510
L_CYS135	-0.093589	-0.000034	-0.093555
L_LEU136	-0.045637	-0.000086	-0.045550
L_ILE137	0.065840	-0.000252	0.066092
L_PHE140	-0.083053	-0.001771	-0.081282
L_ALA144	0.005539	-0.000484	0.006024
L_VAL145	0.457846	0.002764	0.460610
L_VAL147	0.006970	-0.000172	0.007142
L_ALA158	-0.758227	-0.001765	-0.756462
L_VAL160	-0.112710	-0.022644	-0.090067
L_ALA174	0.400048	-0.088890	0.488939
L_ALA175	-0.535874	-0.043566	-0.492308
L_LEU179	-0.000724	-0.000000	-0.000724
E_{total} (kcal/mol)		-22.577685	
